# Engineering stable radicals using photochromic triggers

**DOI:** 10.1038/s41467-020-14798-9

**Published:** 2020-02-18

**Authors:** Xuanying Chen, Wandong Zhao, Gleb Baryshnikov, Michael L. Steigerwald, Jian Gu, Yunyun Zhou, Hans Ågren, Qi Zou, Wenbo Chen, Liangliang Zhu

**Affiliations:** 1 State Key Laboratory of Molecular Engineering of Polymers, Department of Macromolecular Science, Fudan University, Shanghai, 200438 China; 2 Shanghai Key Laboratory of Materials Protection and Advanced Materials in Electric Power, Shanghai University of Electric Power, Shanghai, 200090 China; 3 Division of Theoretical Chemistry and Biology, School of Chemistry, Biotechnology and Health, KTH Royal Institute of Technology, SE-10691 Stockholm, Sweden; 4 Department of Chemistry, Columbia University, New York, NY 10027 USA

**Keywords:** Synthetic chemistry methodology, Photocatalysis, Excited states

## Abstract

Long-standing radical species have raised noteworthy concerns in organic functional chemistry and materials. However, there remains a substantial challenge to produce long-standing radicals by light, because of the structural dilemmas between photoproduction and stabilization. Herein, we present a pyrrole and chloride assisted photochromic structure to address this issue. In this well-selected system, production and stabilization of a radical species were simultaneously found accompanied by a photochemical process in chloroform. Theoretical study and mechanism construction indicate that the designed π-system provides a superior spin-delocalization effect and a large steric effect, mostly avoiding possible consumptions and making the radical stable for hours even under an oxygen-saturated condition. Moreover, this radical system can be applied for a visualized and quantitative detection towards peroxides, such as 2,2,6,6-tetramethylpiperidine-1-oxyl, hydrogen peroxide, and ozone. As the detection relies on a radical capturing mechanism, a higher sensing rate was achieved compared to traditional redox techniques for peroxide detection.

## Abstract (web summary)

Long-standing radical species have raised noteworthy concerns in organic chemistry and but there remains a substantial challenge to produce long-standing radicals by light. Here, the authors demonstrate a stable dithienylethene derived photochromic radical for detection of peroxides and ozone.

## Introduction

Radicals that can be stabilized in chemical systems have played an important role in regulating chemical reactivity and material physical properties^1–3^. By delocalizing spin and blocking subsequent reactions, chemists have developed several molecular structures (e.g. multi-cyano skeletons^4^, tetramethyl azacyclic systems^5^, polythiophenes^6,7^, azulenes^8^, porphyrins^9^, and other extended aromatic compounds^10^) for achieving stable radical species. These systems also show remarkable potential in organic optoelectronics, magnetic materials and magnetic resonance imaging, non-linear optical devices, as well as energy storage^11,12^. Nevertheless, the stable radical generations thus far have largely relied on redox reactions or thermal processes, lacking the convenience from the perspective of material manipulation. In contrast, light stimulus is typically precise and rapid, and can provide contactless spatial and temporal control^13–17^. Although it is popular to employ photon (or photothermal effect) to generate intermediate radicals for reactions, to straightforwardly produce stable radical species by light, which will be in favor of further developing photochemical methodology as well as for steady-state material application, is still challenging but desirable.

To produce stable radicals by light, we need to overcome the dilemmas in chemical design between the photoproduction and the stabilization factors. There are two facts that mostly hamper the above-mentioned hypothesis: (1) unpaired electrons produced upon photoirradiation can easily suffer from ultrafast back pairing relaxation; (2) there is lack of structural characteristics to stabilize radicals in chemical systems even if they are formed. Here we note that the photochromism, as a representative photochemical behavior, which can undergo rapid and efficient photocontrol process accompanied by dramatic π-electron rearrangement to change the apparent color^18–23^, may have potential to fulfill the task. Photochromic materials have received widespread interest in probes, information storage, logic gates, self-assemblies, biological materials, etc^24,25^. During a photochromic process, the exciton can undergo an intermediary transition to an excited state. In this way, it exerts diverse conversion possibilities and can ideally ensure a follow-up coupling with orbitals of nearby molecules. Photochromism can also coexist with radical species in some presupposed magnetic molecular structures, featuring a superior orthogonality in material design^26–29^. On the other hand, relatively stable radicals formed from hexaarylbiimidazole for photochromism were also reported^30–32^. However, to produce even stronger and more stable radical properties for steady-state material application triggered by photochromic process remains difficult, simply because the existed photochromic skeletons lack a sufficient spin-delocalization effect to stabilize the radicals once formed.

**Fig. 1:**
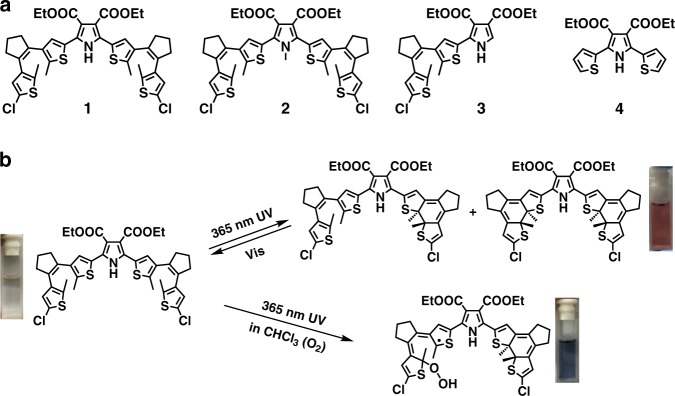
Research outline. **a** Illustration for related chemical structures: compounds 1–4. **b** An outcome of photochromic switching and radical production processes of compound 1 controlled by light in oxygen-saturated chloroform.

Dithienylethene is a typical photochromic moiety enabling a relatively large and planar molecular geometry during photocyclization^33,34^. In another hand, pyrrole and chloride can work as effective assisted groups to stabilize the radicals in large π-conjugated systems^35,36^. We envisage that pyrrole and chloride assisted conjugated structures based on dithienylethenes can provide a superior chemical environment for radical growth by light and radical stabilization by delocalizing spin. Our inspiration origins not only from previous reports on the photochemical feature of dithienylethenes, but also from the fact that the intrinsic properties of dithienylethenes can readily be modulated by rational backbone modification. Therefore, by clamping two chlorinated dithienylethenes into a modified pyrrole center, a sufficiently large conjugated system with photochromic triggers is designed and synthesized (compound 1, see Fig. 1a). To get deeper insight into the intrinsic structural rationality of compound 1, we also extend to more systems by synthesizing a series of reference compounds, namely 2, 3, and 4, for control study (Fig. 1a), from the perspective of regulating the chlorinated dithienylethene and pyrrole. And we eventually find that the compound 1 can perform as we expected by optimization (Fig. 1b).

## Results

### Radical properties upon irradiation

**Fig. 2:**
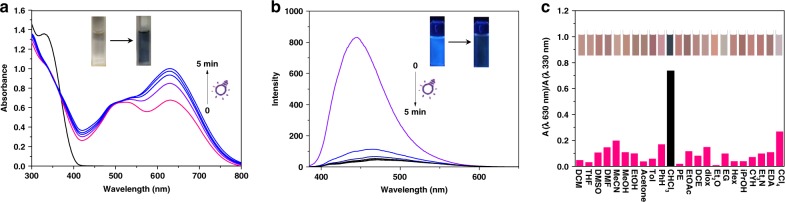
Photochemical properties. **a** UV–vis absorption spectral and **b** fluorescence spectral changes of compound 1 (5.0 × 10^−5^ M) in oxygen-saturated CHCl_3_ solution upon irradiation with 365 nm light at room temperature. **c** The absorption ratio (A_630 nm_/A_330 nm_) of compound 1 in a series of solvents and the corresponding color changes (inset).

UV–vis spectra of compound 1 changed significantly within 1 min upon irradiation with 365 nm light (Fig. 2a), suggesting a high sensitivity and fast response to UV irradiation. A typical absorption band at ~500 nm emerged, indicating a typical photochromic behavior accompanied by the photocyclization of dithienylethene from its ring-open form to ring-closed one. This is further verified by fluorescence quenching (Fig. 2b).

To our surprise, however, an additional absorption band at ~630 nm also appeared upon a continuous irradiation in chloroform, and continued to grow with the prolongation of the irradiation time till ~5 min (Fig. 2a). To further investigate the unusual dual band characteristic, UV–vis spectra of compound 1 were measured in a series of different solvents (the details including the solvent abbreviations are listed in Supplementary Fig. 1). It was found that only one band centered at around 500 nm emerged after UV irradiation with a colorless solution converted to pink, except in chloroform. High performance liquid chromatography (HPLC) showed that two new peaks formed simultaneously upon the photoirradiation (Supplementary Fig. 2), suggesting that both dithienylethene units of compound 1 participate in the chemical process with different efficiency in these solvents except chloroform. To exclude the influence of residue acid in chloroform on the corresponding properties of compound 1, we first determine the purity of chloroform solvents by gas chromatography (GC), suggesting the purity of the solvents is quite high without any impurity (Supplementary Figs. 3 and 4). Then UV–vis spectra were measured in different alkali-treated and acid-treated chloroform under the same conditions, respectively. The absorption changes in these treated chloroform solvents show the same tendency as used in the common chloroform (Supplementary Figs. 5–7). Thus, we conclude that the gradient color from pink to blue in chloroform should be attributed to an additional mechanism, rather than a common photochromism.

**Fig. 3:**
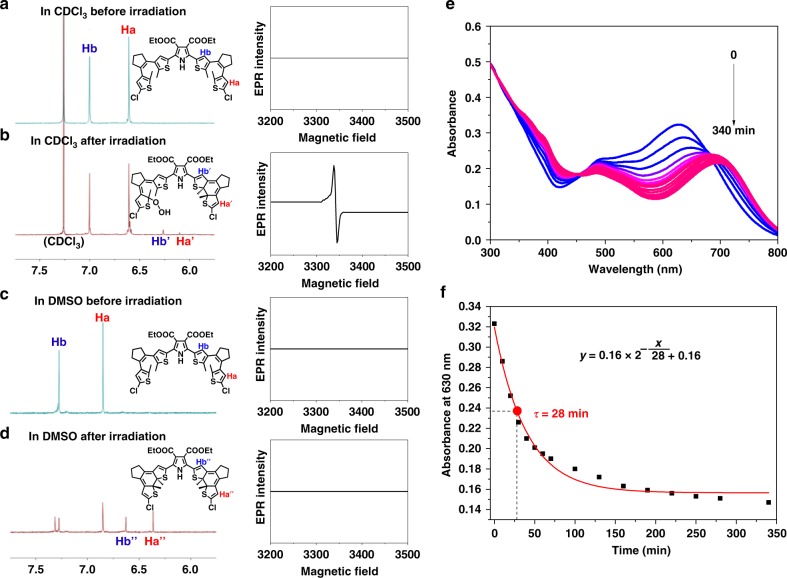
Characterization for the radical species. **a**–**d** ^1^H NMR spectra of compound 1 in CDCl_3_ and DMSO-*d*_6_ and the corresponding EPR spectra without and with irradiation of 365 nm light at 25 °C, respectively. **e** UV–vis absorption spectral changes and **f** the decay curve of compound 1 (1.0 × 10^−5^ M) in oxygen-saturated CHCl_3_ solution after withdrawing the photoirradiation.

A radical formation upon photochemical triggering can then be inferred, since a radical can easily result in a significant absorption shift^1–3,11,12^. The absorbance ratio (A_630 nm_/A_330 nm_, Fig. 2c) reflects the relative yield of the possible radical species, suggesting that such a process in chloroform is outstandingly efficient. As traditional photoswitching compounds, the cyclization and cycloreversion processes of the dithienylethene can be well monitored by ^1^H NMR spectra^37,38^. In our case, compound 1 in DMSO-*d*_6_ connects to a standard photocyclization characterization with obvious ^1^H NMR shifts (Fig. 3 and Supplementary Fig. 9), similar to the typical photochromism of dithienylethenes^33,34^. In contrast, the ^1^H NMR spectra of compound 1 in CDCl_3_ only show tiny signal change before and after UV irradiation (see Fig. 3). Besides, we see from Supplementary Fig. 8 that a small chemical shift change on proton and the linewidth is slightly broadened upon irradiation of compound 1 in CDCl_3_. We reason that the lack of a huge chemical shift change and the line broadening is due to the localization of a possible radical on the carbon, hence it has minimal contact effect on the proton in the nearby groups. Due to the short electron relaxation time as well as the fast rotation of these groups, the persudo-contact effect in the hyperfine interaction can be decoupled and shows little effect on the proton spectrum^39,40^. These results strengthened the fact that a radical process may occur within chloroform, which revealed a relatively lower sensitivity in ^1^H NMR change but competitively weakened the ratio of the photocyclization (see a tiny ^1^H NMR signal of protons Ha’ and Hb’ in Fig. 3b).

Electron paramagnetic resonance (EPR) measurements provided direct evidence of the radical formation and suggested that radicals with a magnetic field strength at 3200–3400 mT were generated in CDCl_3_ solution after UV irradiation (Fig. 3b). In contrast, the EPR spectra did not show radical signals in DMSO-*d*_6_ solution after UV irradiation (Fig. 3c, d). These results feature a solvent-dependence of yielding the radical. It is worth noting that such a radical could be easily detected by EPR even without any radical trapping agent, suggesting that they are quite stable. The lifetime of the radicals is further determined to be hours by a decay curve based on the absorbance at 630 nm in chloroform under an oxygen-saturated condition (Fig. 3e, f).

### Proposed radical production mechanism

**Fig. 4:**
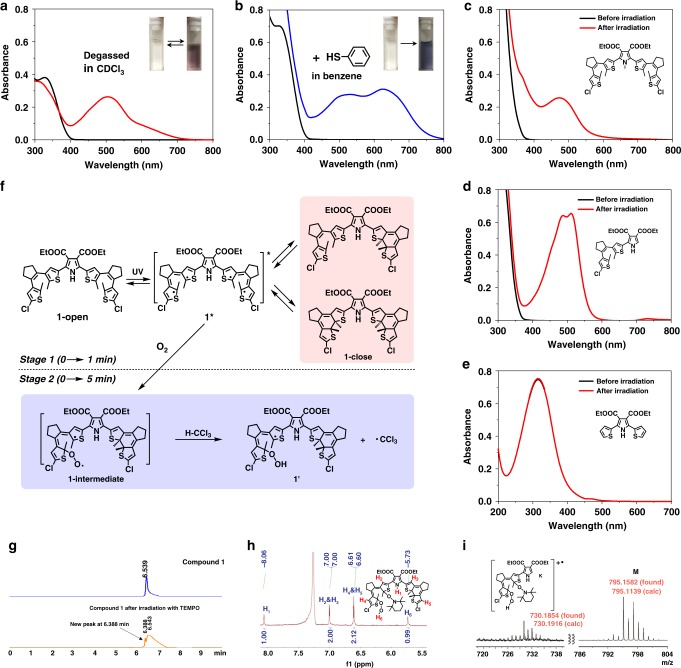
Mechanism study on the radical species. **a** UV–vis absorption spectral changes of compound 1 (1.0 × 10^−5^ M) upon irradiation with 365 nm light for 5 min in degassed CHCl_3_. Inset: the corresponding solution color change. **b** UV–vis absorption spectral changes of compound 1 (1.0 × 10^−5^ M) with 10 equiv. of thiophenol in benzene upon irradiation with 365 nm light for 5 min at 25 °C. Inset: the corresponding photographic images upon irradiation with 365 nm light for 5 min. UV–vis absorption spectral changes of compounds 2 (**c**), 3 (**d**), 4 (**e**) in CHCl_3_ (1.0 × 10^−5^ M) upon irradiation with 365 nm light. **f** A proposed radical generation process in molecule 1. **g** HPLC spectra of compound 1 (above) and compound 1 after irradiation with TEMPO (below) at 25 °C under following conditions: Agilent C18 column, acetonitrile-water (6:4, v/v) as mobile phase, the detection wavelength of 254 nm. **h** Partial ^1^H NMR spectrum of isolated TEMPO-coupled compound corresponding to the new peak at retention time of 6.388 min in HPLC. **i** MS (MALDI-TOF) of photo-irradiated compound 1 in oxygen-saturated CHCl_3_ solution with addition of 10 equiv. of TEMPO.

The reference compounds 2, 3, and 4 were synthesized to get insight into the mechanism of the radical production. The UV–vis spectra of the reference compounds 2, 3 and 4 are shown in Fig. 4c–e, and here we can only observe the photochemical process on compounds 2 and 3 rather than compound 4, signifying the importance of the existence of the dithienylethene unit. That means the “photochromic triggers” are necessary. Similarly, ^1^H NMR peaks of compounds 2 and 3 in CDCl_3_ did not change remarkably upon irradiation with 365 nm light (Supplementary Figs. 10 and 11). The EPR spectra of compounds 2 and 3 also indicate that radicals exist in CDCl_3_ with 365 nm irradiation (Supplementary Fig. 12). However, the absorbance ratio (A_630 nm_/A_330 nm_) of compounds 2 and 3 is relatively low, suggesting a less efficient production or existence of radicals in compounds 2 and 3 than in compound 1. Therefore, the big structural regulation in compound 1 is definitely helpful for the production and stabilization of the photo-triggered radical species. We can even see that there is no EPR signal when the blank chloroform solvent was illuminated under the same conditions (Supplementary Fig. 12), suggesting that the radical signal indeed originates from the studied structures rather than from possible free radical residue in the solvents or other disruptors.

According to a relevant report^41^, the EPR signal position near the magnetic field strength of 3350 indicates that there exist oxygen species in the system. The blue solution cannot be obtained for compound 1 in degassed chloroform upon UV irradiation (Fig. 4a), further proving that the radical formation in our case requires the participation of oxygen. Such a control study indicates that for further ensuring the stabilization of the possibly yielded radicals, a dehydrogenation effect by oxygen coupling was involved for the aid of our experiments. On the other hand, a solution of compound 1 with 10 equiv. of thiophenol in benzene could also turn to blue with UV irradiation (Fig. 4b), similar to the same absorption change with that in oxygen-saturated chloroform, featuring a dehydrogenation effect on the peroxide-based radical formation in the presence of an activable hydrogen donor. And thus, we can propose that the excited state 1* (which can also be referenced from a typical photochromic process, see Fig. 4f) as a diradical firstly forms upon photoexcitation with UV light. Followed by this photochromic trigger, in the presence of O_2_, 1* can be favorable to combine with oxygen to give an intermediate. The intermediate can then immediately extract a relatively active hydrogen atom (for instance, H-CCl_3_ and H-SC_6_H_5_ in our case) to form a peroxide-based monoradical species 1′, corresponding to the blue color of the solution which is much more stable. While compound 1 exhibited an identical signal position in EPR and absorption spectra with that of compound 3, we can conclude that the radical species is a mono-hydroperoxide one (like the structure showed in Fig. 4f), leaving the other dithienylethene still photocyclizable (see the tiny ^1^H NMR signal shifted in Fig. 3 and Supplementary Fig. 8 for details, respectively).

We believe that the above-mentioned mechanism is reasonable because CHCl_3_ is the relatively most effective hydrogen donor upon irradiation^42,43^. In this way, this process can be perfectly demonstrated by the presence of an even strong hydrogen donor (thiophenol, see Fig. 4b). The radical species is still difficult to be directly detected by mass spectrometry simply because of its oxygen and CHCl_3_ dependence. Therefore, only unreacted molecular ion peaks can be found (see Supplementary Fig. 13 for comparison). However, an indirect approach can be employed by the addition of 2,2,6,6-tetramethylpiperidine-1-oxyl (TEMPO) to covalently couple with the monoradical species to prove this issue. Key fragment peaks (e.g. *m/z* = 730.1854) upon the coupling structure were observed in Fig. 4i (see also the illustration for the coupling and fragment analysis in Supplementary Fig. 14), the high-resolution value of which showed a very close agreement with that calculated (mass error < 9 ppm). Besides, preparative HPLC was employed to isolate the adduct of photo-irradiated compound 1 coupled with TEMPO (from the new compound signal with the retention time of 6.388 min, see details showed in Fig. 4g and Supplementary Fig. 15). As shown in the ^1^H NMR spectrum (see Fig. 4h), several characteristic peaks were observed, including the pyrrole proton, thiophene protons and peroxide proton. These results for the adduct show good agreement with the mechanism we proposed. In addition, we emphasize that another possible mechanism regarding a participation of Cl radical can be ruled out, since a common photochromic phenomenon was also observed in carbon tetrachloride (see Fig. 2c and Supplementary Fig. 1v). These control studies further supported the suggested route as shown in Fig. 4f.

As photochromism is always an incomplete photoconversion process, we are unfortunately unable to employ single crystal XRD to precisely characterize the radical structure. We instead carried out a calculation study to verify our assumption and to analyze the stability. As can be seen from the spin-density analysis (Supplementary Fig. 16 and Supplementary Table 1), the radical can be assigned as a π-type strongly delocalized one. This fact actually explains its high stability. We can see the prevailing contribution to the spin-density plots from the S-containing dithienylethene branches and also from the central pyrrole ring, suggesting the necessity of an alternating structure. The single-occupied molecular orbitals (SOMOs) are completely responsible for the observed spin-density patterns. Here the spin-density plots can be considered as the square of SOMO orbitals whereas the contribution from other alpha-spin orbitals is found to be negligible. Regarding the calculated isotropic g-factor, a considerable positive deviation of the average g-factor from that for the free electron can be observed: 2.004 vs. 2.002. By analyzing the g-tensor components (Supplementary Table 1), we see that the XX and YY components for the radical are almost the same and vary in the range 2.004–2.006, while the ZZ component is much smaller (around 2.004). Such an anisotropy of the g-tensors is typical for stable π-type radicals^44–46^. In our case, the radical is placed in the XY plane, while Z axis is perpendicular to this plane. On the other hand, some theoretical calculations on ^1^H NMR (Supplementary Table 2) and the absorption (Supplementary Fig. 17) of the proposed radical species were also performed and summarized in Supplementary Information. The calculations show good agreement in the signal positions with the experimental results for compound 1 and its photostationary state. The photostability of the photocyclized skeleton is good, as a relatively obvious photobleaching can only be observed after 24 h upon continuous irradiation (Supplementary Fig. 18).

### Application for visualizing ozone detection

The UV–vis absorption spectra and color of the radical species of compound 1 are sensitive to peroxides, such as TEMPO and hydrogen peroxide. As shown in Supplementary Fig. 19, after adding TEMPO and hydrogen peroxide, respectively, UV–vis absorption spectra of compound 1 at the photostationary state in CHCl_3_ changed with the disappearance of the characteristic peak at 630 nm, due to the radical capturing effect. Therefore, the photostationary state of compound 1 in CHCl_3_ can be used as a detector for peroxide with naked-eye recognition. To better monitor the qualitative sensing ability, we measured UV–vis absorption spectral changes of compound 1 at the photostationary state in CHCl_3_ in the presence of increasing concentrations of TEMPO and hydrogen peroxide, respectively, and plotted the corresponding absorbance at 630 nm with respect to the concentrations of TEMPO and hydrogen peroxide (Supplementary Figs. 20 and 21). It is found that the absorbance at 630 nm could be well fitted to the concentration of TEMPO and hydrogen peroxide, respectively, suggesting that compound 1, as a photochromic probe, could achieve the practical calibration and quantitative determination of peroxide.

**Fig. 5:**
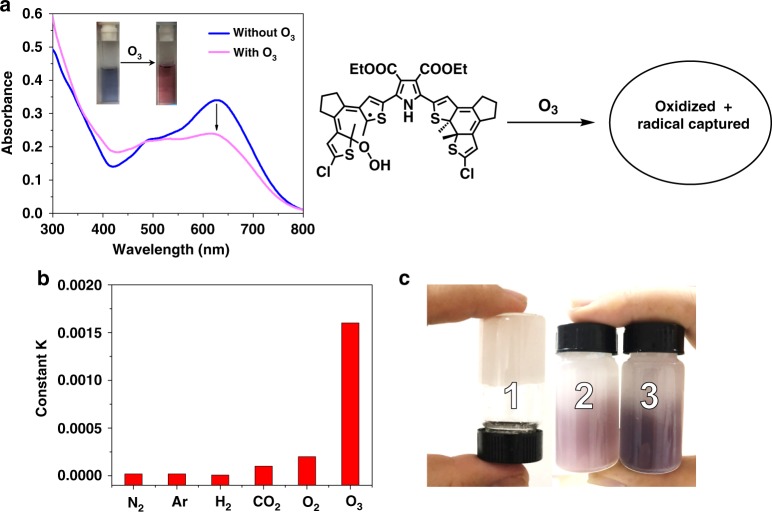
Application for visualizing ozone detection. **a** UV–vis absorption spectral changes of compound 1 (1.0 × 10^−5^ M) in CHCl_3_ solution at the photostationary state in the presence of O_3_ at 25 °C. Inset: the corresponding solution color change. **b** The rate of absorbance changes at 630 nm of photo-irradiated compound 1 in CHCl_3_ solution after bubbling with various types of gas. **c** A gel of compound 1 containing chloroform: 1. the original state; 2. O_3_ bubbled photostationary state; 3. the photostationary state.

Therefore, we employed this material to construct an easy-to-use probe for the detection of ozone by colorimetry. Peroxides, particularly ozone, are important oxidants that have many industrial and consumer applications related to oxidation, whereas an excessive production of peroxides makes them potent respiratory hazards and pollutants near the ground level^47,48^. When ozone flow generated from the ozonator (see Supplementary Fig. 22) was immersed into the CHCl_3_ solution of compound 1 at the photostationary state, the blue solution converted to deep red within 20 s along with an absorption change (Fig. 5a). In this way, it can serve as a rapid detector for ozone with naked-eye visualization. As presented in Fig. 5b, the change rate of compound 1 with O_3_ was the fastest one among the various types of gas (see also the fitting process in detail in Supplementary Fig. 23), featuring that a possible radical capturing mechanism played key roles, not just an oxidation process. Besides, we prepared the gel 1 from compound 1 by blending the polyvinyl chloride with the corresponding chloroform solution as shown in Fig. 5c. The apparent color could turn to blue from colorless after UV irradiation similarly. Upon O_3_ bubbling, the blue gel quickly turned to pink gel (from 3 to 2). This suggests that the gel based on compound 1 at the photostationary state can work well as a portable O_3_ detector. We can calculate the O_3_ volume from the flow rate per unit time, thus to achieve a quantitative ozone detection by this material. Such a detection with a radical capturing mechanism ensures a higher sensing rate compared to traditional redox techniques for peroxide detection.

## Discussion

In summary, a unique stable radical formation strategy triggered by photochromism has been demonstrated. The molecular design of a pyrrole and chloride assisted photochromic structure has been proved to play the key role. Our compound shows a two-stage response to UV light, due to the generation of an unusual radical other than the traditional photocyclization. It is proposed that the dehydrogenation effect helps to stabilize the electron departure upon photoexcitation to yield a peroxide-based species. Furthermore, the structural design for the conjugated structure provides a spin-delocalization effect and a large steric effect, making the radical stable under oxygen-saturated conditions for hours. It is argued that such a radical species can be manipulated as radical trapping agents to detect peroxides by colorimetry, including TEMPO, hydrogen peroxide, and ozone. These detections can be quantitative and portable. The presented photochromism triggered stable radical formation may pave a way for future development of photochemical methodologies as well as the photomanipulation of advanced optoelectronic materials.

## Methods

### Chemicals and characterization

1,2-bis(5-chloro-2-methyl-3-thienyl) cyclopentene^49^ and compound 4^50^ were prepared and purified according to literature procedures, respectively. All other reagents were commercially available and used without further treatment. The synthesis details of the intermediates T1, T2, T3, T4, and T5 were shown in Supplementary Information. Thin-layer chromatography (TLC) analyses were performed on silica-gel plates, and flash chromatography was conducted by using silica-gel column packages purchased from Qing-dao Haiyang Chemical Company, China. ^1^H NMR and ^13^C NMR spectra in CDCl_3_ or DMSO-*d*_*6*_ were recorded on Brucker AM-400 spectrometers with tetramethylsilane (TMS) as the internal standard. High-resolution mass spectrometry (HRMS) were recorded on a Waters LCT Premier XE spectrometer with methanol as solvent and a Matrix Assisted Laser Desorption Ionization-Time of Flight (MALDI-TOF) Mass Spectrometer (5800), respectively. UV–vis absorption spectra were recorded on a Shimadzu 1800 spectrophotometer, while the fluorescent emission spectra were taken with a Shimadzu RF-5301 PC; both spectrophotometers were standardized. HPLC were recorded on a Shimadzu LC-20A with Agilent C-18 column.

### Synthesis of compound 1

Under nitrogen atmosphere, to a solution of compound T1 (0.370 g, 1.0 mmol) and freshly prepared T2 (3.04 mmol) in degassed toluene (20.0 mL) was added Pd(PPh_3_)_4_ (0.173 g, 0.150 mmol) at room temperature, and the resulting mixture was purged with nitrogen for 30 min and then stirred overnight at 100 °C. After cooling to room temperature, the mixture was concentrated and purified by column chromatography (silica gel, CH_2_Cl_2_/petroleum ether 4:1, v/v) to give the compound 1 (0.528 g, 66%) as a pale-yellow solid. ^1^H NMR (400 MHz, CDCl_3_): δ 1.32 (t, *J* = 6.0 Hz, 6H), 1.87 (s, 6H), 2.02–2.09 (m, 10H), 2.74 (t, *J* = 8.0 Hz, 4H), 2.80 (t, *J* = 8.0 Hz, 4H), 4.28 (q, *J* = 8.0 Hz, 8H), 6.61 (s, 2H), 7.00 (s, 2H), 8.09 (s, 1H). ^13^C NMR (100 MHz, CDCl_3_): δ 14.19, 14.28, 22.92, 38.28, 38.31, 60.85, 125.13, 126.83, 127.31, 127.71, 127.99, 133.35, 134.38, 134.92, 135.05, 135.88, 136.37, 164.64. HRMS (ESI+): [M + Na]^+^ calcd. for C_40_H_39_NO_4_NaS_4_Cl_2_, m/z: 818.1037; found, 818.1035.

### Synthesis of compound 2

Under nitrogen atmosphere, to a solution of T3 (0.467 g, 1.22 mmol) and freshly prepared T2 (3.66 mmol) in degassed toluene (25.0 mL) was added Pd(PPh_3_)_4_ (0.212 g, 0.183 mmol) at room temperature, and the resulting mixture was purged with nitrogen for 30 min and then stirred overnight at 100 °C. After cooling to room temperature, the mixture was concentrated and purified by column chromatography (silica gel, CH_2_Cl_2_/petroleum ether 4:1, v/v) to give the compound 2 (0.722 g, 71%) as a pale-yellow solid. ^1^H NMR (400 MHz, CDCl_3_): δ 1.21 (t, *J* = 6.0 Hz, 6H), 1.91 (s, 6H), 2.03–2.10 (m, 10H), 2.73 (t, *J* = 6.0 Hz, 4H), 2.79 (t, *J* = 6.0 Hz, 4H), 3.21 (s, 3H), 4.18 (q, *J* = 8.0 Hz, 4H), 6.58 (s, 2H), 6.69 (s, 2H). ^13^C NMR (100 MHz, CDCl_3_): δ 14.11, 22.96, 32.74, 38.12, 38.19, 60.51, 116.19, 125.08, 126.05, 126.91, 129.30, 131.34, 133.20, 134.25, 135.07, 135.18, 135.50, 137.35, 164.44. HRMS (ESI+): [M + Na]^+^ calcd. for C_41_H_41_NO_4_NaS_4_Cl_2_, m/z: 832.1193; found, 832.1172.

### Synthesis of compound 3

Under nitrogen atmosphere, to a mixture of T4 (0.145 g, 0.50 mmol), aqueous potassium carbonate (10.0 mL, 1.7 M) and Pd(PPh_3_)_4_ (0.058 g, 0.05 mmol) in degassed THF (20.0 mL) was added the solution of freshly prepared compound T5 (0.75 mmol) using a syringe at room temperature, and the resulting mixture was purged with nitrogen for 30 min and then stirred overnight at reflux. After cooling to room temperature, the mixture was extracted with CH_2_Cl_2_ (20.0 mL × 3), combined organic layers were washed with saturated NaCl solution (20.0 mL) and dried over anhydrous Na_2_SO_4_, filtrated, and concentrated. The residue was purified by column chromatography (silica gel, ethyl acetate/petroleum ether 1:3, v/v) to give the compound 3 (0.130 g, 49%) as a yellow solid. ^1^H NMR (400 MHz, CDCl_3_): δ 1.33 (t, *J* = 6.0 Hz, 6H), 1.86 (s, 3H), 1.97–2.05 (m, 5H), 2.71–2.78 (m, 4H), 4.30 (q, *J* = 8.0 Hz, 4H), 6.58 (s, 1H), 6.96 (s, 1H), 7.31 (s, 1H), 8.44 (s, 1H). ^13^C NMR (100 MHz, CDCl_3_): δ 14.18, 14.32, 22.86, 38.35, 38.42, 60.30, 61.19, 116.97, 123.43, 125.10, 126.78, 126.91, 127.81, 127.95, 133.28, 134.30, 134.80, 134.95, 135.66, 136.00, 163.66, 165.72. HRMS (ESI+): [M + Na]^+^ calcd. for C_25_H_26_NO_4_NaS_2_Cl, m/z: 526.0903; found, 526.0898.

### Computational details

The structure of the compounds 1 and its closed forms were optimized at the DFT level of theory using the B3LYP^51,52^ functional and 6-31G(d)^53^ basis set. The structure of the open-shell structure 1′ was optimized by the same method but using the spin-unrestricted DFT formalism. The parameters of the g-tensor for the unpaired electron in the radical 1′ were calculated by gauge-independent atomic orbital (GIAO) method^54^ using the B3LYP functional and 6-311++G(d,p)^55–57^ basis set. The solvent effect was accounted through the polarizable continuum model (PCM)^58^ by using CHCl_3_ as a model solvent (*ε* = 4.711). Using the ground state optimized geometries, the energies and intensities of singlet-singlet and doublet-doublet electronic transitions were estimated by TD-DFT method^59^. The spin purity of doublet-doublet electronic transitions was concluded from the <S^2^> eigenvalues close to 0.75. All the calculations have been carried out using the Gaussian16 software^60^. The isosurfaces for molecular orbitals and spin densities were generated using the Chemissian software^61^.

## Supplementary information

Supplementary Information

## Data Availability

The data that support the findings of this study not included in the supplementary information document are available from the corresponding author upon reasonable request.
