# Supplementary material for: Engineering stable radicals using photochromic triggers
Source: Nat Commun. 2020 Feb 18;11:945. doi: 10.1038/s41467-020-14798-9 (PMC7028928; doi:10.1038/s41467-020-14798-9)
Supplement: Supplementary file 1 — Supplementary Information [file 41467_2020_14798_MOESM1_ESM.pdf]

Supplementary Information

Engineering stable radicals using photochromic triggers

Chen et al.

Table of Contents

No.	Contents	Page
1	Supplementary Figures 1–37	3–26
2	Supplementary Tables 1–2	27–28
3	Supplementary Methods	29–31
4	Supplementary Note	32–33
5	Supplementary References	34

Supplementary Figures

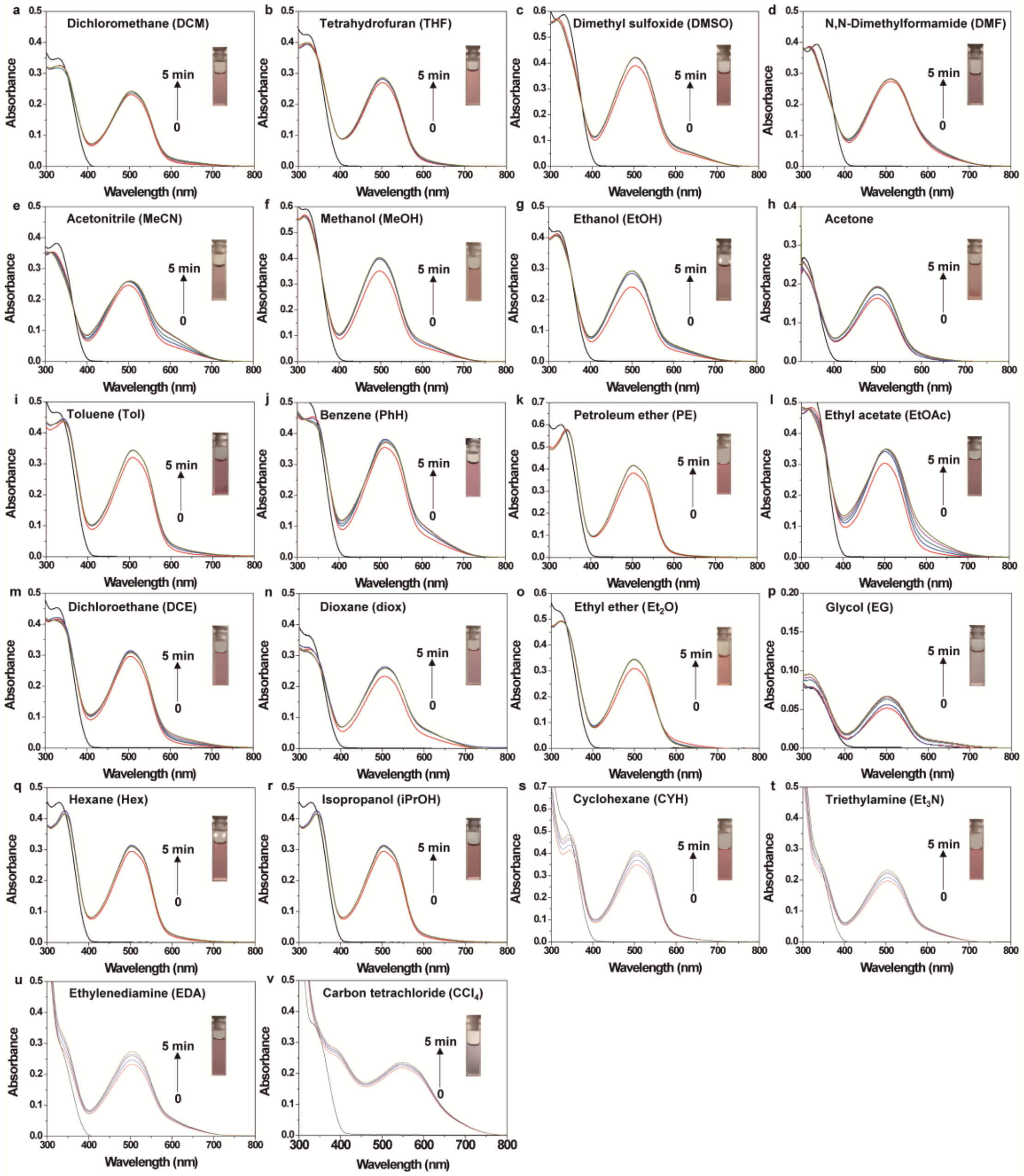

Supplementary Figure 1. Photochemical properties of compound **1** in different solvents. UV–vis absorption spectral changes of compound **1** (1.0×10^{-5} M) in a series of solvents upon irradiation with 365 nm light for 5 min at 25 °C, respectively. Insets: the corresponding photographic images upon irradiation with UV light for 5 min, respectively.

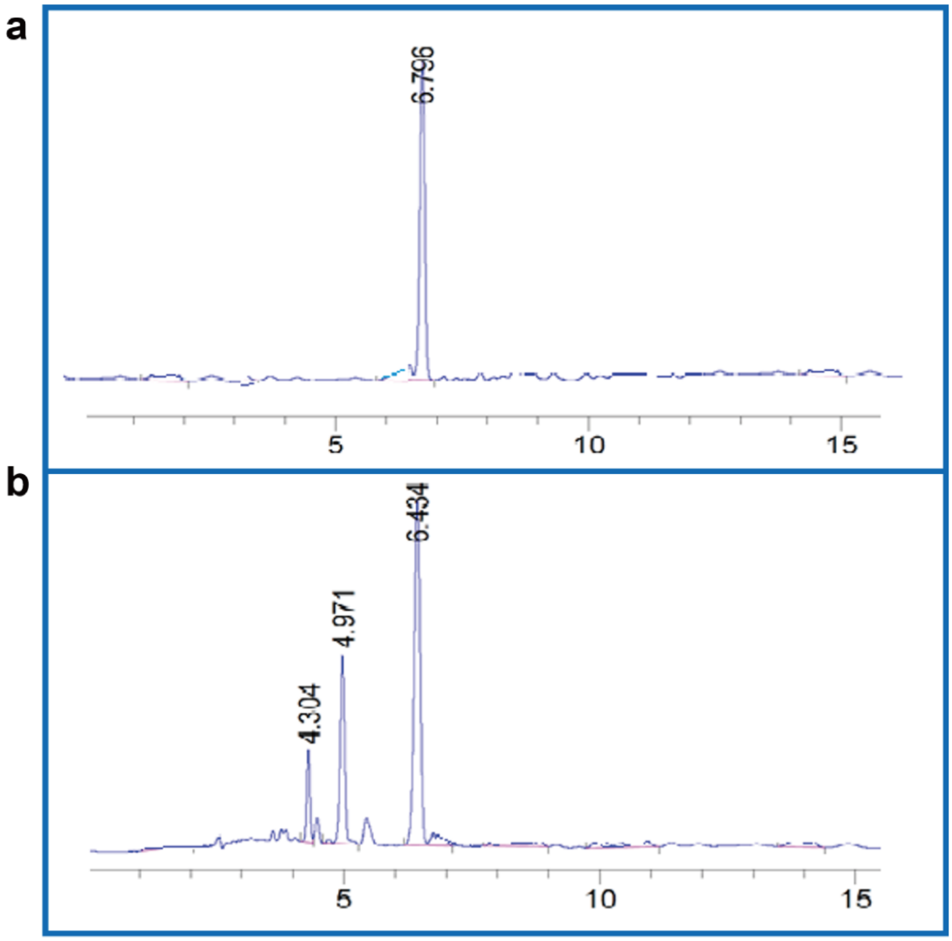

Supplementary Figure 2. HPLC analysis for the photocyclization process of compound **1**. The HPLC spectra of compound **1** (1.0×10^{-4} M) in acetonitrile without (**a**) and with (**b**) irradiation of 365 nm light at 25 ^{o}C under the following conditions: Agilent C18 column, acetonitrile-water (8:2, v/v) as mobile phase, the detection wavelength of 254 nm.

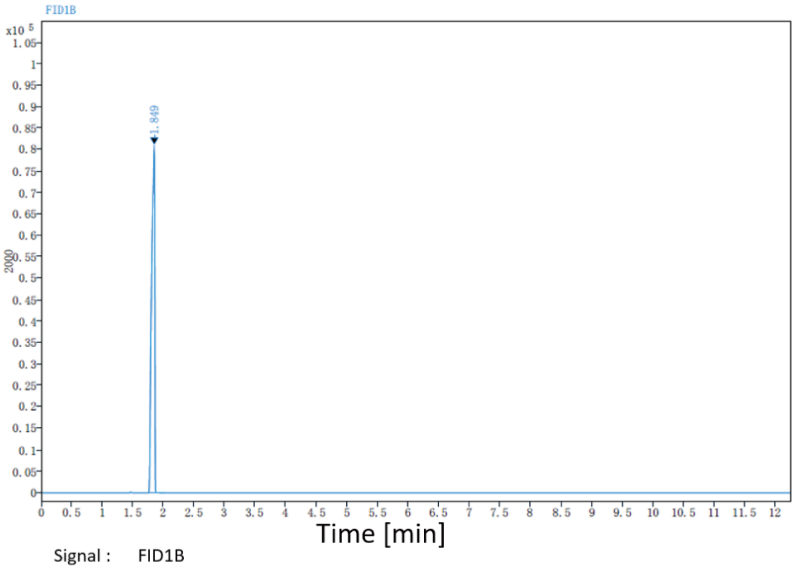

Signal : FID1B

Time [min]	Peak area	Peak height	Peak area (%)
1.849	284858.3361	81117.0395	100.0000
Total	284858.3361		

Supplementary Figure 3. GC analysis for chloroform. Gas chromatographic (GC) spectrum of chloroform used for the tests. GC conditions: Agilent DB-5 column; 300 ^{o}C as inlet temperature; 50 ^{o}C (1 min) to 290 ^{o}C at 30 ^{o}C/min (hold 3 min) for a total run time of 12 min as oven program.

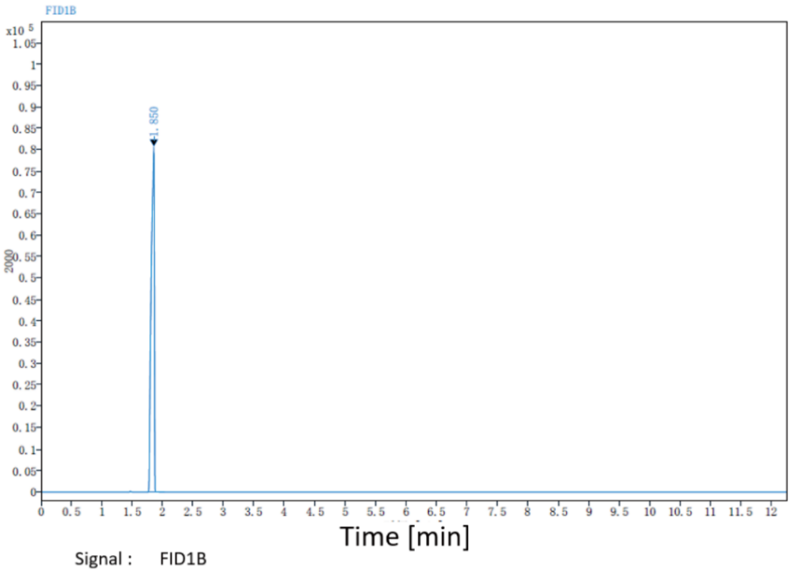

Signal : FID1B

Time [min]	Peak area	Peak height	Peak area (%)
1.850	283407.4872	80770.7377	100.0000
Total	283407.4872		

Supplementary Figure 4. GC analysis for deuterated chloroform. Gas chromatographic (GC) spectrum of deuterated chloroform used for the tests. GC conditions: Agilent DB-5 column; 300 ^{o}C as inlet temperature; 50 ^{o}C (1 min) to 290 ^{o}C at 30 ^{o}C/min (hold 3 min) for a total run time of 12 min as oven program.

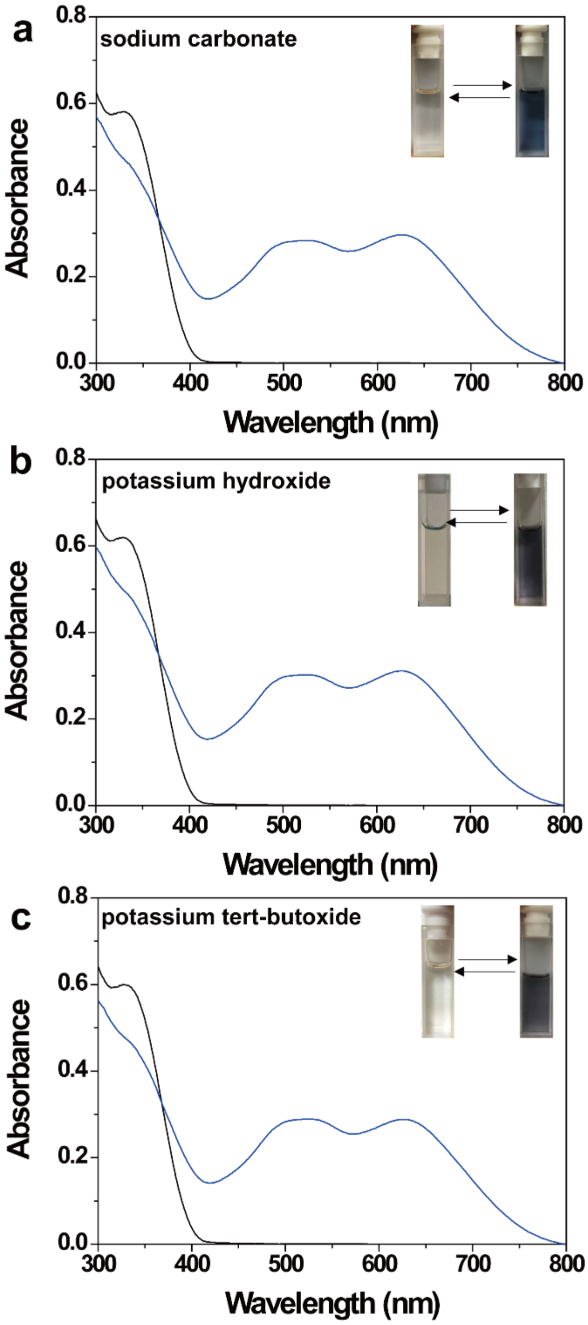

Supplementary Figure 5. Photochemical properties of compound **1** in alkali-treated solvents. UV–vis absorption spectral changes of compound **1** (1.0×10^{-5} M) upon irradiation with 365 nm light for 5 min in $CHCl_3$ treated with sodium carbonate (**a**), potassium hydroxide (**b**) and potassium tert-butoxide (**c**), respectively. Inset: the corresponding solution color change.

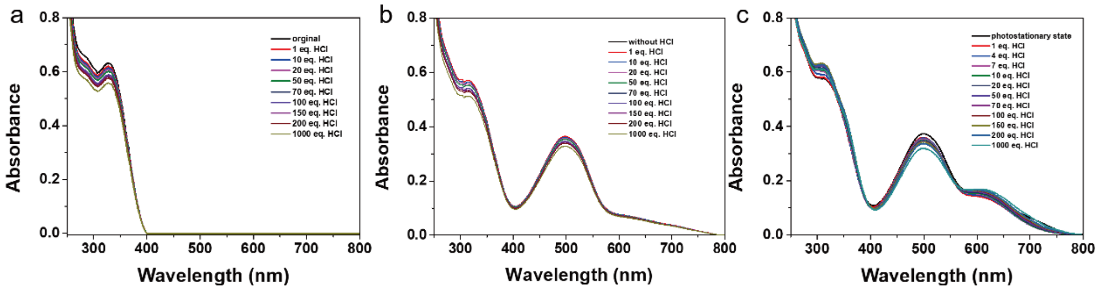

Supplementary Figure 6. Photochemical properties of compound **1** in acetonitrile with concentrated hydrochloric acid (HCl). (**a**) UV–vis absorption spectral changes of compound **1** (1.0×10^{-5} M) in acetonitrile with different amounts of concentrated HCl at 25 °C. (**b**) UV–vis absorption spectral changes of compound **1** (1.0×10^{-5} M) in acetonitrile with different amounts of concentrated HCl upon irradiation with 365 nm light for 5 min at 25 °C. (**c**) UV–vis absorption spectral changes of compound **1** (1.0×10^{-5} M) in acetonitrile at the photostationary state with different amounts of concentrated HCl at 25 °C.

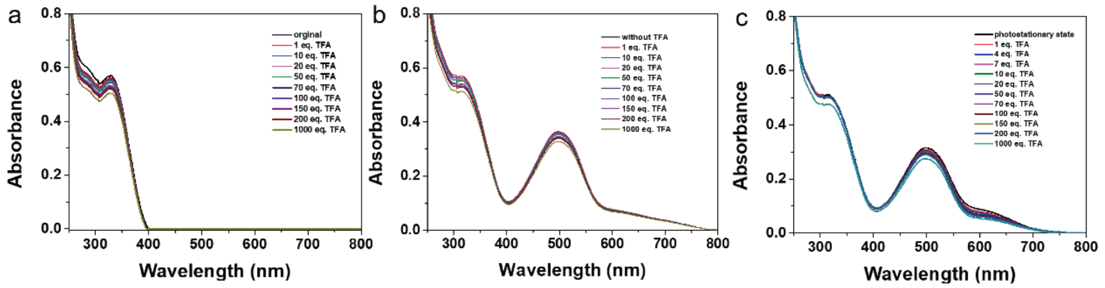

Supplementary Figure 7. Photochemical properties of compound **1** in acetonitrile with trifluoroacetic acid (TFA). (**a**) UV–vis absorption spectral changes of compound **1** (1.0×10^{-5} M) in acetonitrile with different amounts of TFA at 25 °C. (**b**) UV–vis absorption spectral changes of compound **1** (1.0×10^{-5} M) in acetonitrile with different amounts of TFA upon irradiation with 365 nm light for 5 min at 25 °C. (**c**) UV–vis absorption spectral changes of compound **1** (1.0×10^{-5} M) in acetonitrile at the photostationary state with different amounts of TFA at 25 °C.

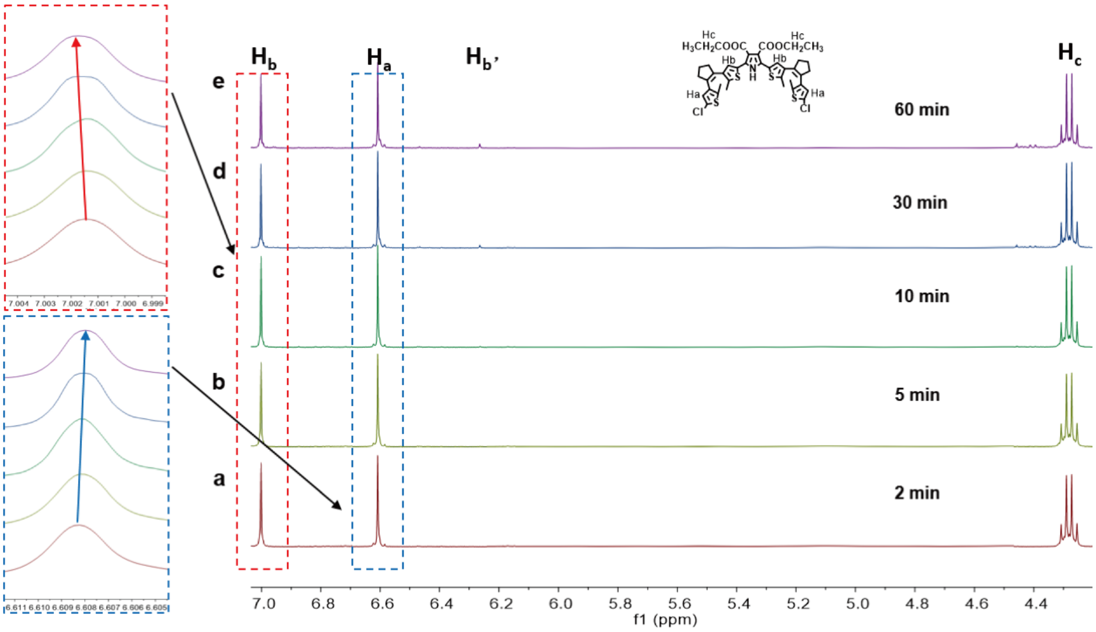

Supplementary Figure 8. ^{1}H NMR spectra of compound **1** in $CDCl_3$ upon irradiation. From bottom to top: the solution under 365 nm light irradiation for 2 min (**a**), 5 min (**b**), 10 min (**c**), and 30 min (**d**), 60 min (**e**). Although we can observe the photocyclization characterization upon the indicative of the emergence of Ha' and Hb' signal, the ^{1}H NMR spectra of compound **1** in $CDCl_3$ only show a tiny signal change before and after UV irradiation (see Fig. 3 and Supplementary Fig. 8, respectively). These results further imply that a radical process may occur within this solvent, which competitively weakened the ratio of a standard photocyclization. On the other hand, the radical moiety with unpaired electrons makes both proton signals (H_a and H_b as shown in Supplementary Fig. 8) assigned to two thiophene groups shifted slightly to upfield and downfield, respectively, along with the slight line broadening in the ^{1}H NMR spectroscopy. It may be reasonable that the radical located in the thiophene moiety attached to pyrrole unit because of the better radical stability stemmed from larger conjugation effect in comparison with that of another thiophene moiety. And thus, corresponding ^{1}H NMR signal change further confirmed the molecular structure proposed in Fig. 4f.

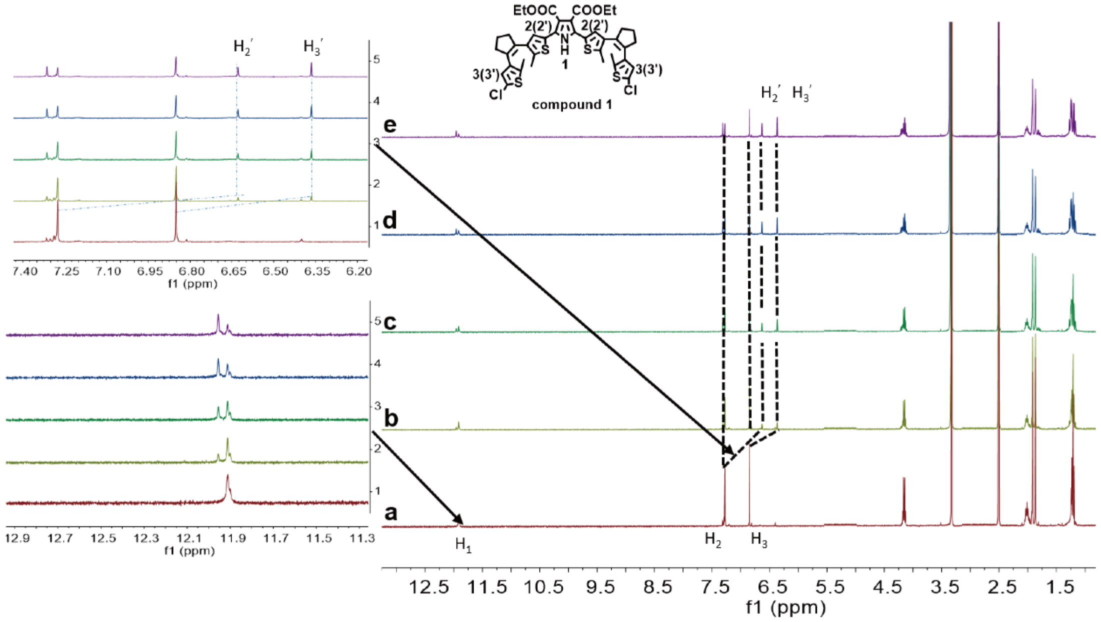

Supplementary Figure 9. ^{1}H NMR spectra of compound **1** in DMSO-d_6 upon irradiation. From bottom to top: original solution (**a**); the solution under 365 nm light irradiation for 8 min (**b**), 16 min (**c**), 24 min (**d**), and 32 min (**e**).

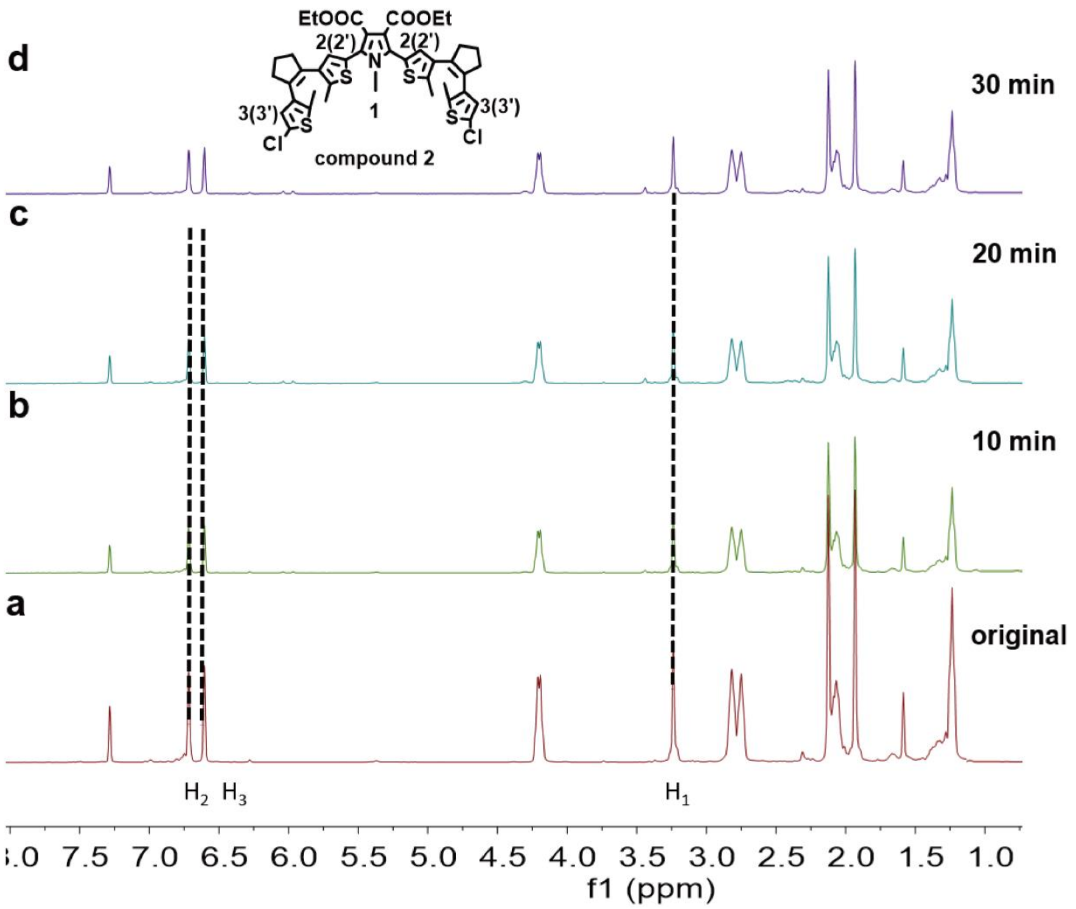

Supplementary Figure 10. ^{1}H NMR spectra of compound **2** in $CDCl_3$ upon irradiation. From bottom to top: original solution (**a**); the solution under 365 nm light irradiation for 10 min (**b**), 20 min (**c**), and 30 min (**d**).

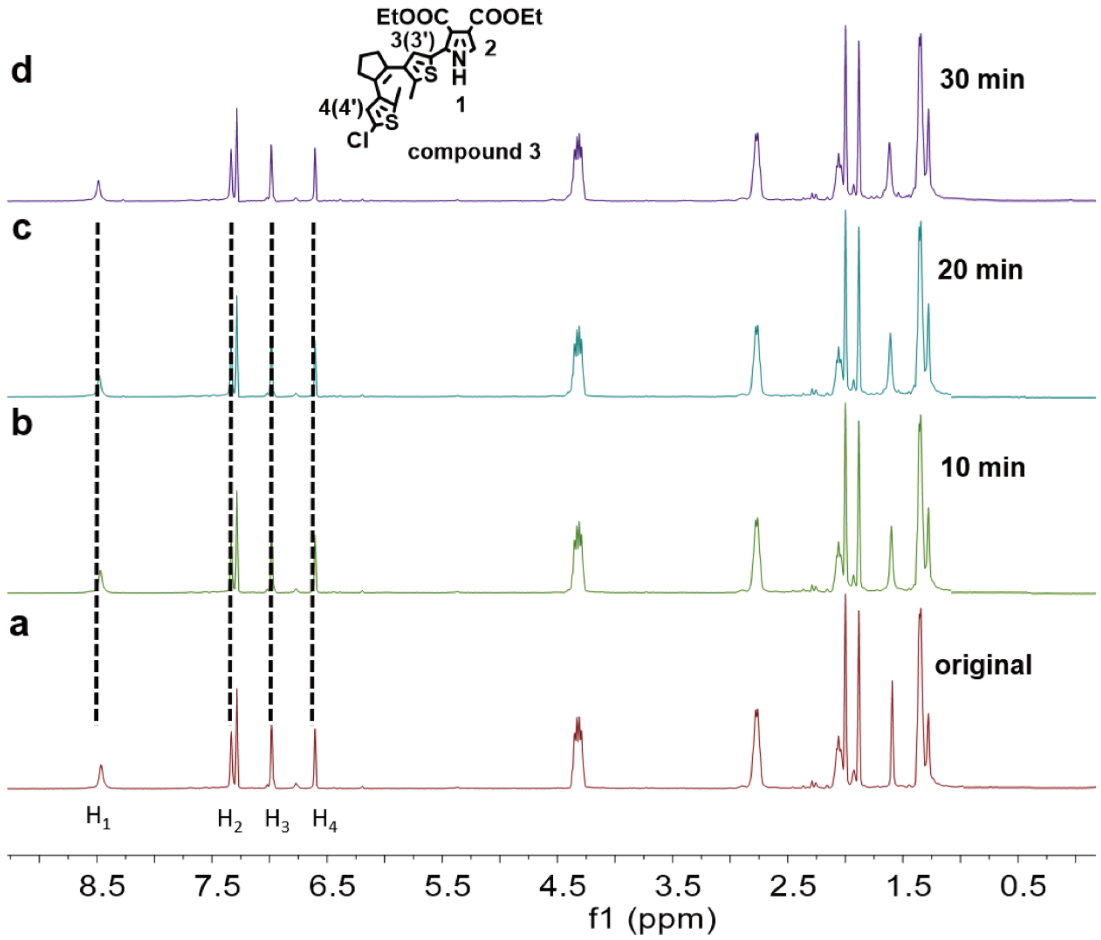

Supplementary Figure 11. ^{1}H NMR spectra of compound **3** in $CDCl_3$ upon irradiation. From bottom to top: original solution (**a**); the solution under 365 nm light irradiation for 10 min (**b**), 20 min (**c**), and 30 min (**d**).

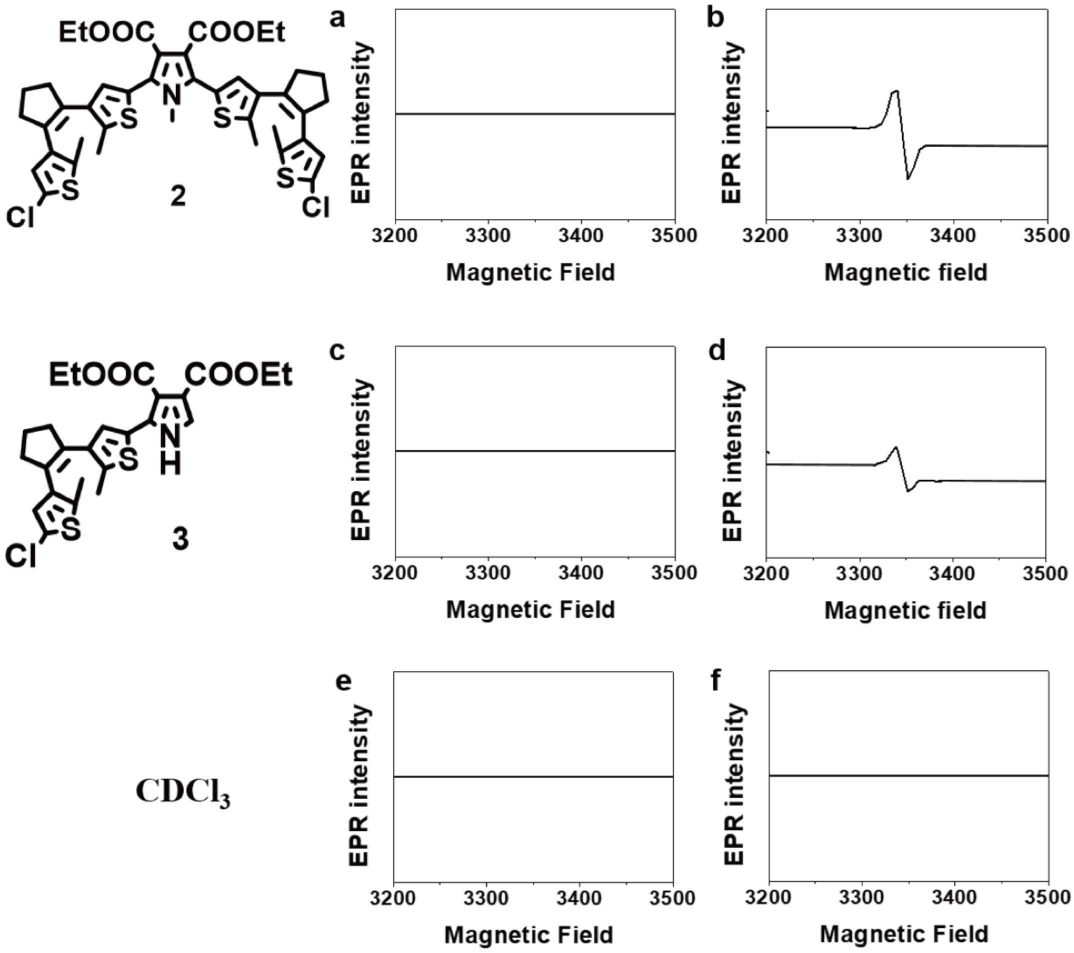

Supplementary Figure 12. EPR spectra of reference compounds. EPR spectra of compound **2** in $CDCl_3$ without (**a**) and with (**b**) irradiation of 365 nm light at 25 °C, respectively. EPR spectra of compound **3** in $CDCl_3$ without (**c**) and with (**d**) irradiation of 365 nm light at 25 °C, respectively. EPR spectra of $CDCl_3$ without (**e**) and with (**f**) irradiation of 365 nm light at 25 °C, respectively.

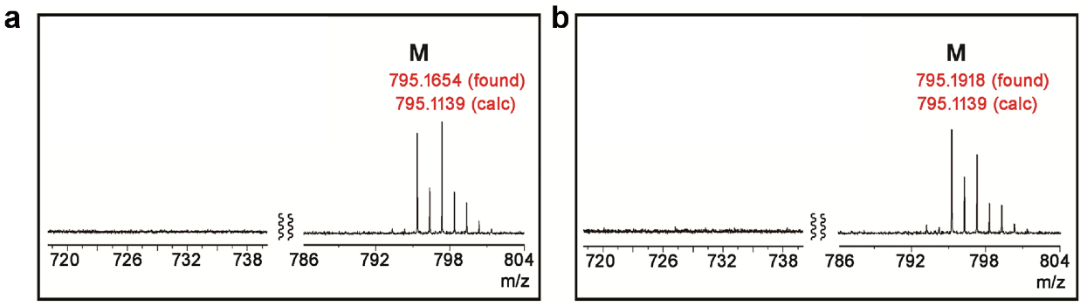

Supplementary Figure 13. MS analysis for compound **1**. MS (MALDI-TOF) of compound **1** in the initial state (**a**), after irradiation with 365 nm light for 5 min (**b**), respectively.

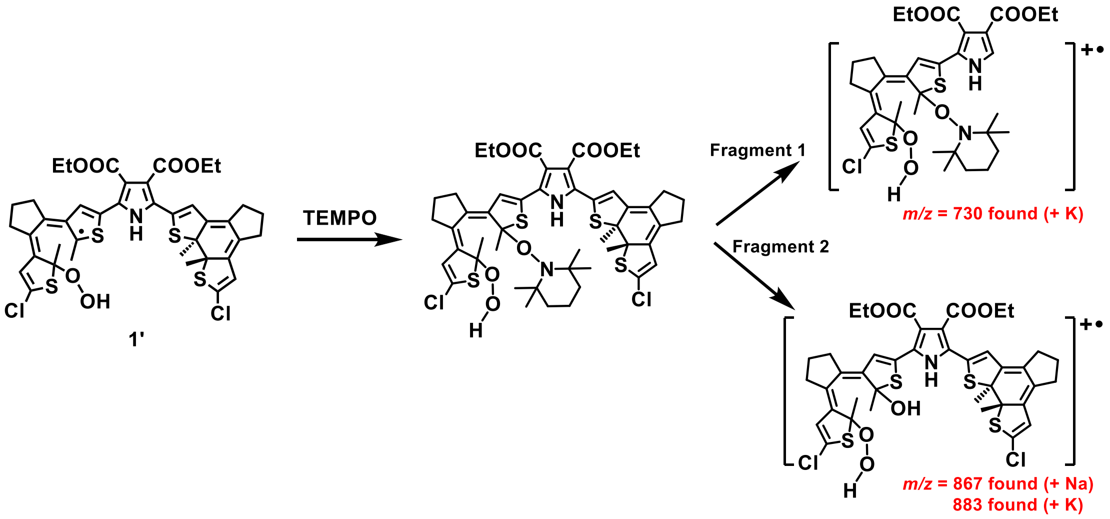

Supplementary Figure 14. MS analysis for photo-irradiated compound **1** with TEMPO. Illustration for the coupling of the formed radical species **1'** with TEMPO and some key fragment signals found in MALDI-TOF-MS. We did not find the whole molecular ion peak due to the bulk molecule that is difficult to survive upon ionization.

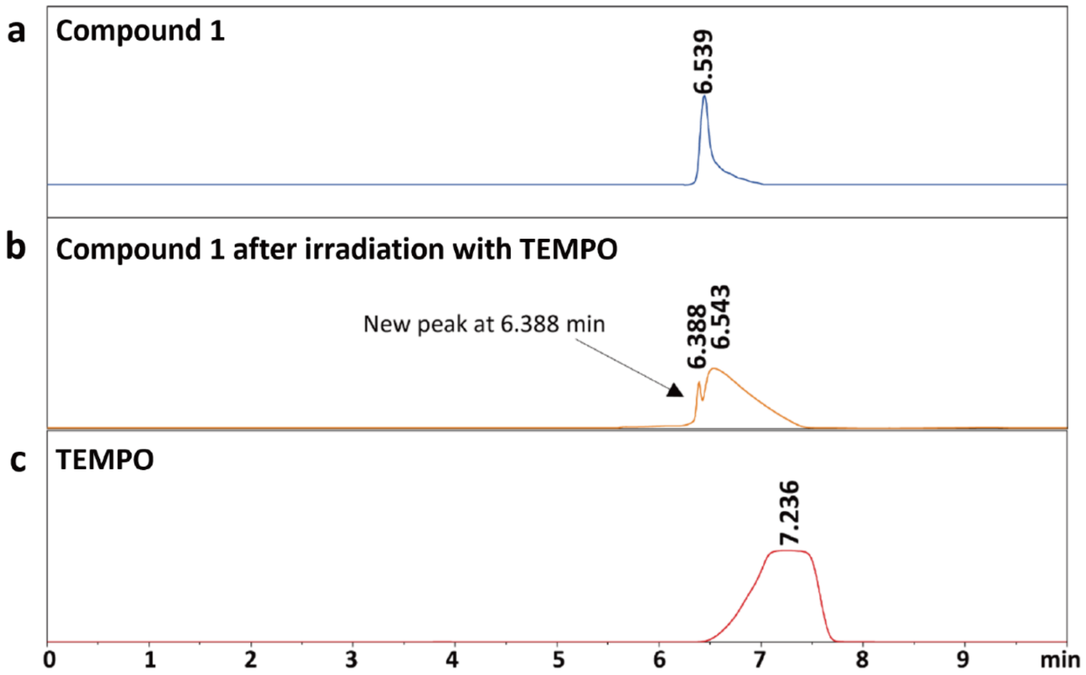

Supplementary Figure 15. HPLC analysis for photo-irradiated compound **1** with TEMPO. HPLC spectra of compound **1** (**a**), compound **1** after irradiation with TEMPO (**b**) and pure TEMPO (**c**) at 25 ℃ under following conditions: Agilent C18 column, acetonitrile-water (6:4, v/v) as mobile phase, the detection wavelength of 254 nm.

a

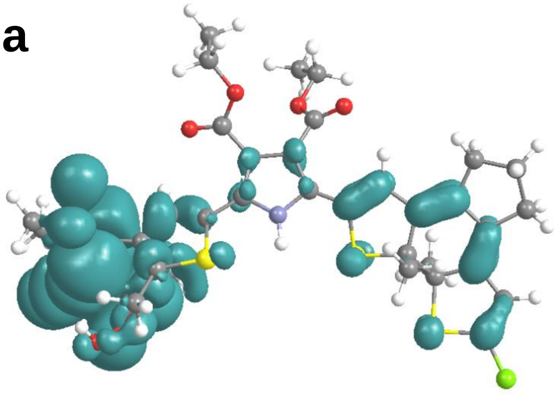

Spin. Dens. (**1**)

b

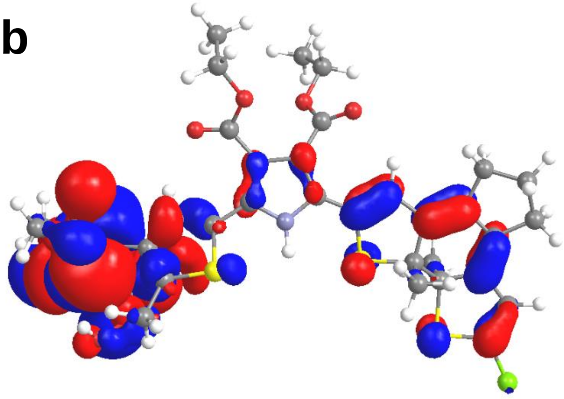

α-SOMO (**1**)

Supplementary Figure 16. Molecular modeling of compound **1**. Theoretically calculated spin-density (**a**) and SOMO (**b**) wavefunction for radical of compound **1** (the control value of isosurfase is 0.01 a.u.).

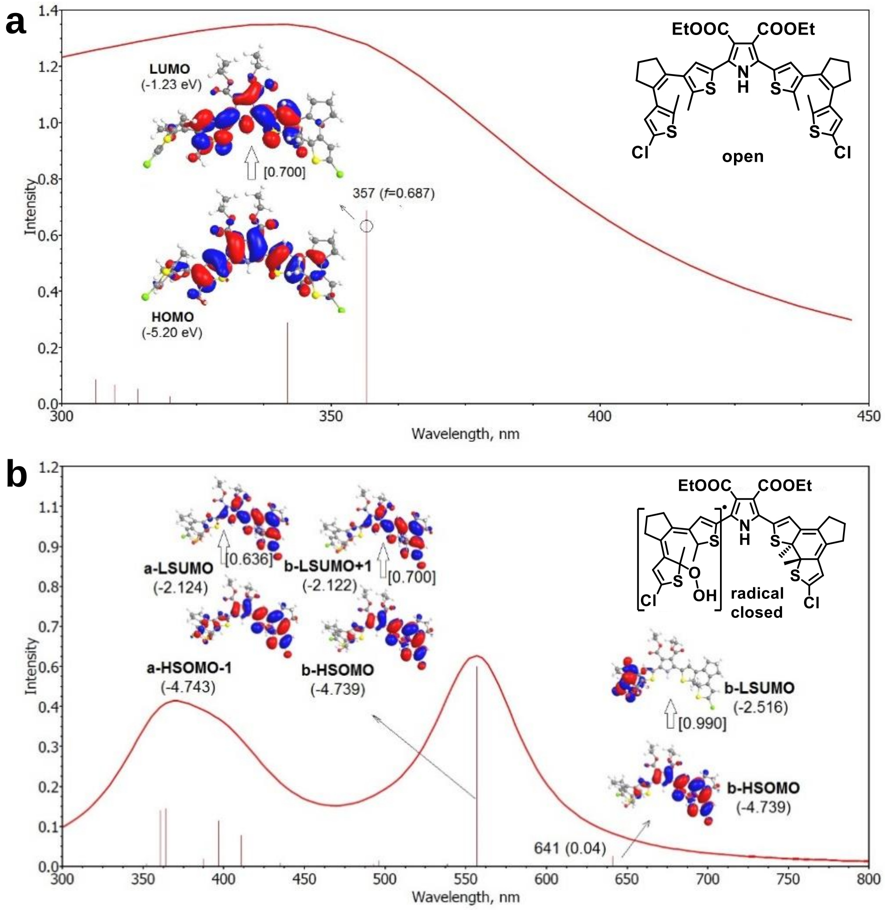

Supplementary Figure 17. Computational absorption spectra of compound **1** in different forms. (PCM)/TD-DFT/B3LYP/6-31G(d) calculated absorption spectra of compound **1** at the different stages of proposed mechanism in Fig. 4f, corresponding to the spectra of initial form (**a**) and radical form (**b**), respectively. The values in brackets correspond to the weight of a particular configuration. MO energies are given in eV as well as energies of selected vertical transitions.

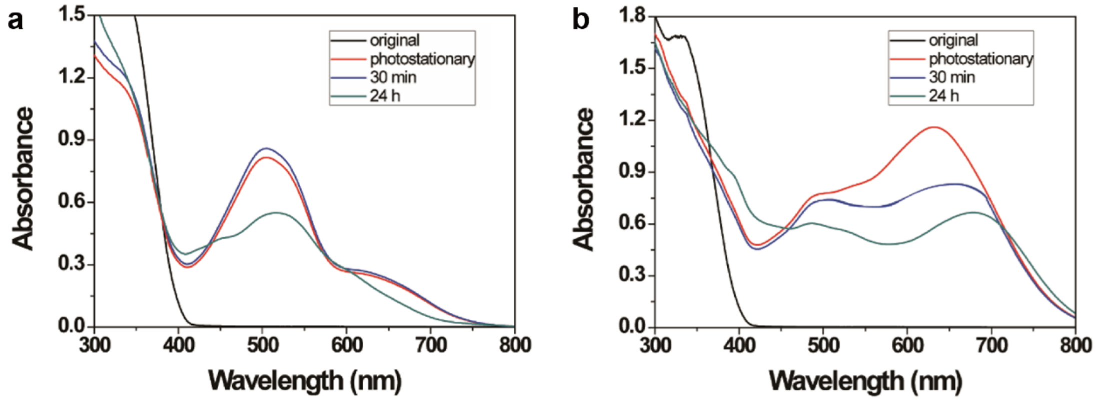

Supplementary Figure 18. Photo-stability and photobleaching study of compound **1**. UV–vis absorption spectral changes of compound **1** at photostationary state in (**a**) CH_2Cl_2 (5.0×10^{-5} M) and (**b**) oxygen-saturated $CHCl_3$ (3.5×10^{-5} M) upon continuous irradiation with white light, respectively.

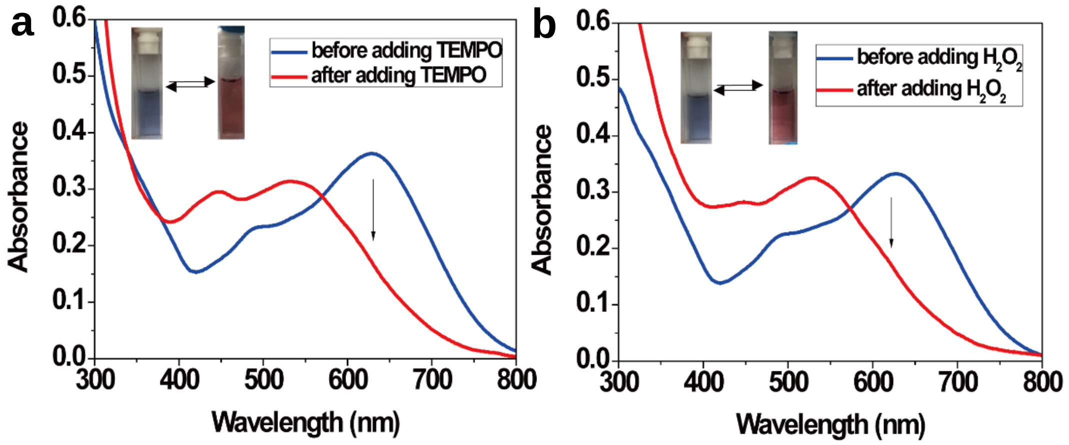

Supplementary Figure 19. Photochemical properties of compound **1** in $CHCl_3$ with peroxides. UV–vis absorption spectral changes of compound **1** (1.0×10^{-5} M) in $CHCl_3$ at the photostationary state with the addition of (**a**) TEMPO; (**b**) H_2O_2, respectively. Inset: the corresponding solution color change.

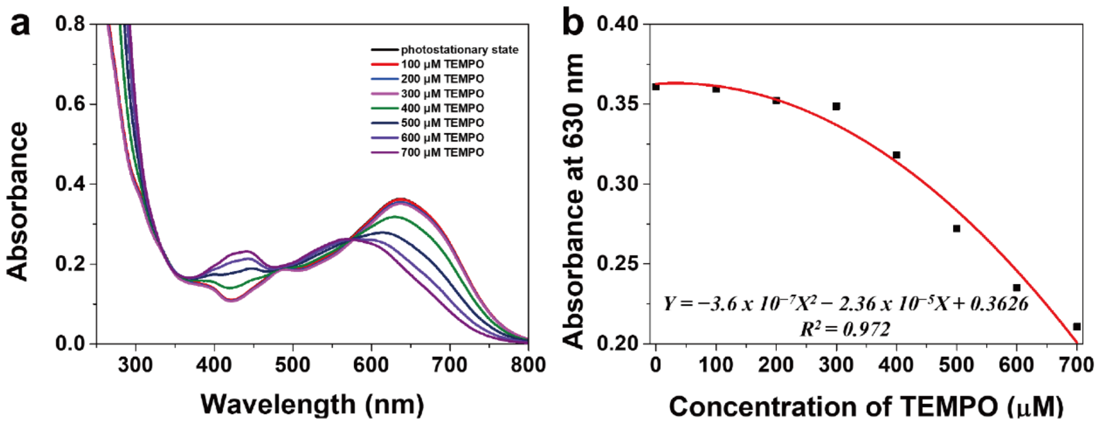

Supplementary Figure 20. Quantitative determination of compound **1** for TEMPO. (**a**) UV–vis absorption spectral changes of compound **1** (1.0×10^{-5} M) in $CHCl_3$ at the photostationary state in the presence of increasing concentrations of TEMPO ($0–7.0\times10^{-4}$ M) at 25 ℃. (**b**) The corresponding absorbance at 630 nm as a cubic function of TEMPO concentration from 0 to 7.0×10^{-4} M with a coefficient $R^2 = 0.972$.

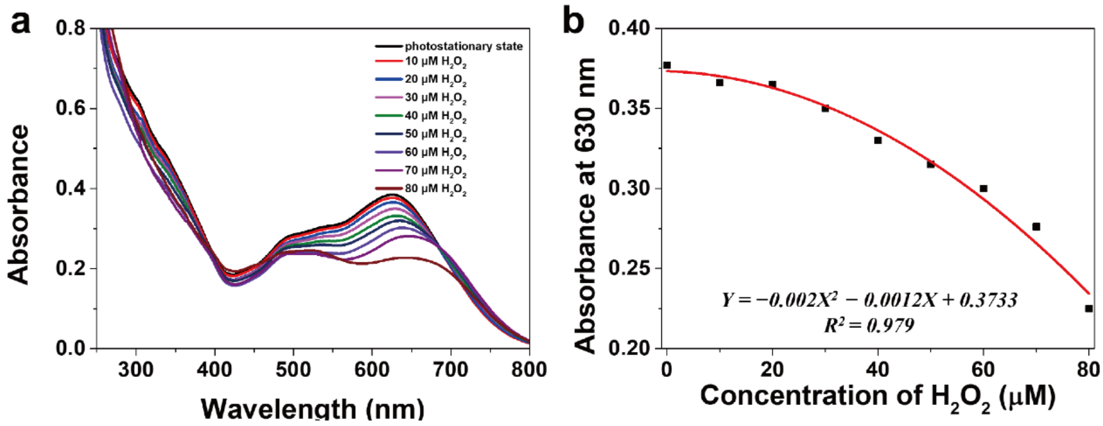

Supplementary Figure 21. Quantitative determination of compound **1** for H_2O_2. (**a**) UV–vis absorption spectral changes of compound **1** (1.0×10^{-5} M) in $CHCl_3$ at the photostationary state in the presence of increasing concentrations of H_2O_2 ($0–8.0\times10^{-5}$ M) at 25 ℃. (**b**) The corresponding absorbance at 630 nm as a quadratic function of H_2O_2 concentration from 0 to 8.0×10^{-5} M with a coefficient $R^2 = 0.979$.

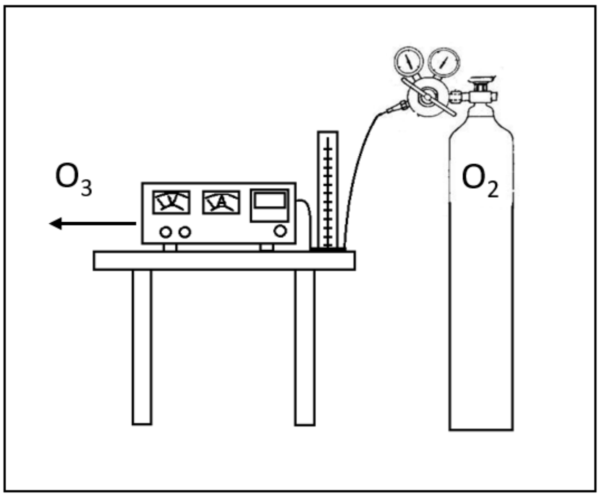

Supplementary Figure 22. Experimental set-up. The schematic diagram of ozonator.

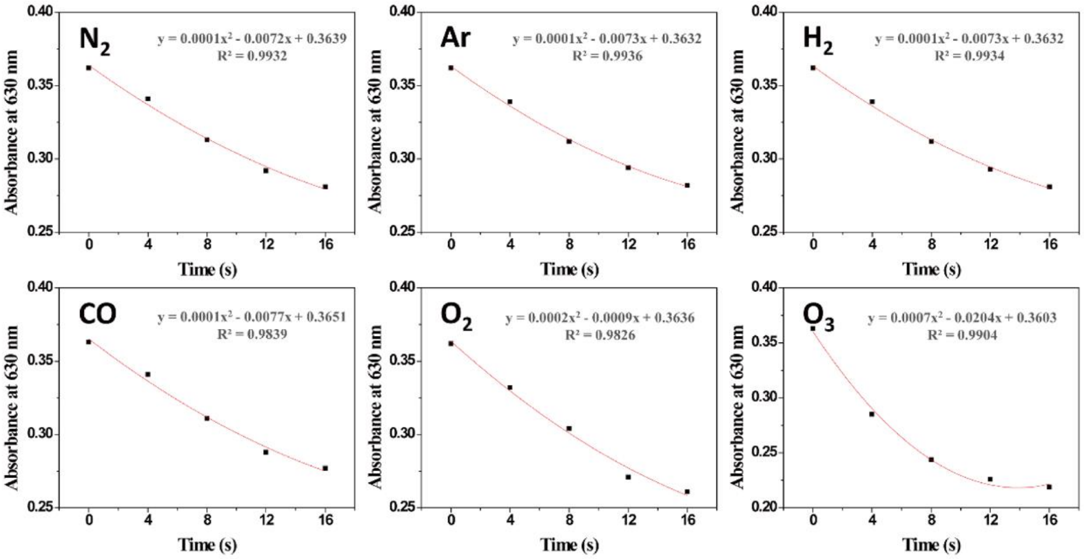

Supplementary Figure 23. The fitting processes of compound **1** to various gases. The fitting results of the absorbance changes at 630 nm of photo-irradiated compound **1** in $CHCl_3$ after bubbling with various types of gas. Compound **1** was illuminated to the photostationary state by 365 nm light, then we bubbled various gases into it and tested its absorption spectral changes at 630 nm, respectively. The data of the spectral changes was quadratically fitted to calculate its change rate constant K that was used to distinguish the sensing ability of compound **1** to various gases.

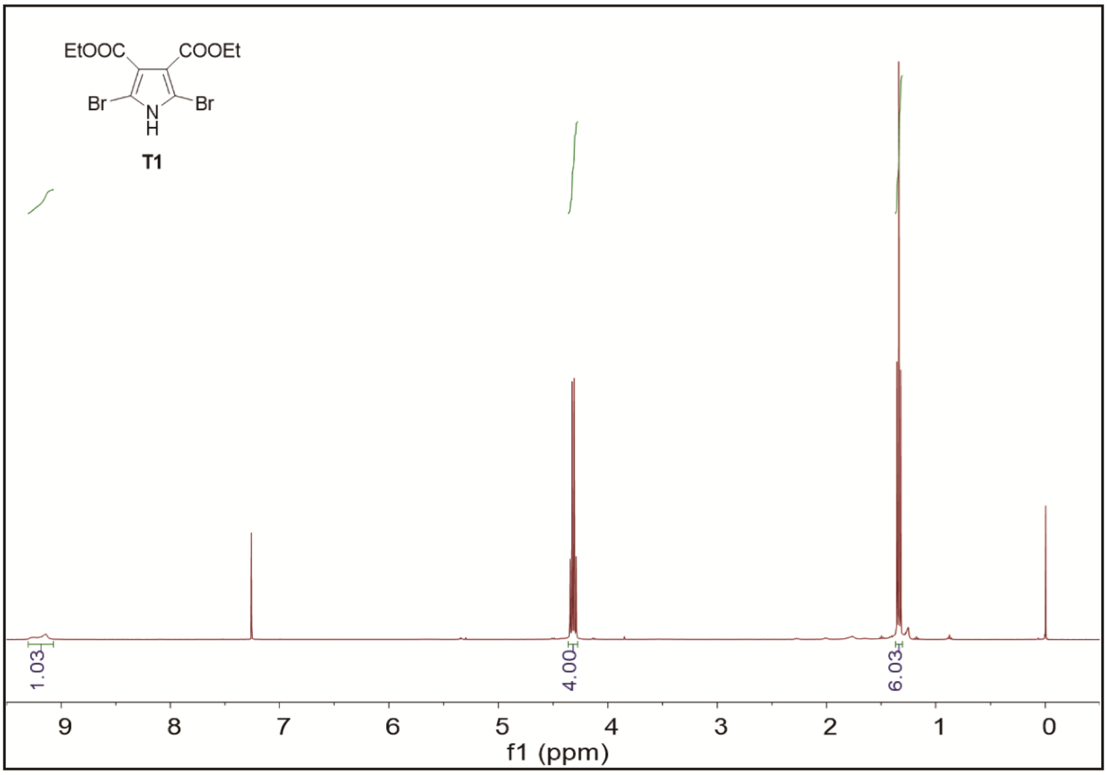

Supplementary Figure 24. ^{1}H NMR (400 MHz, $CDCl_3$) spectrum of compound **T1**.

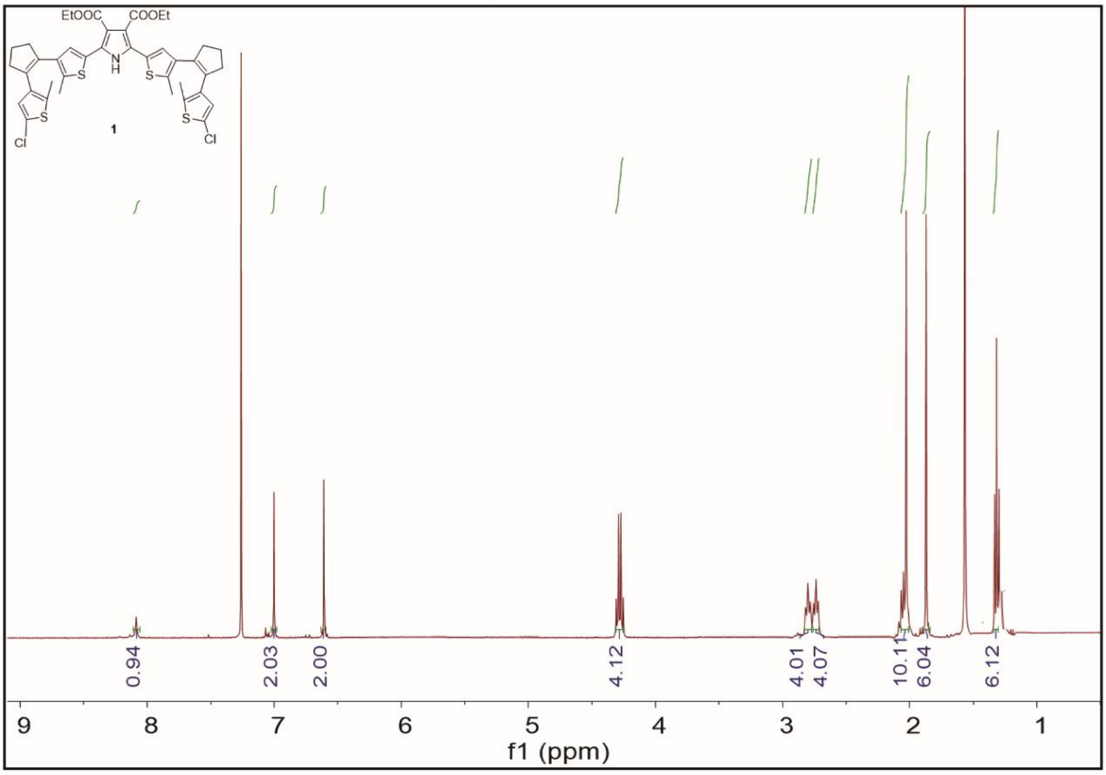

Supplementary Figure 25. ^{1}H NMR (400 MHz, $CDCl_3$) spectrum of compound **1**.

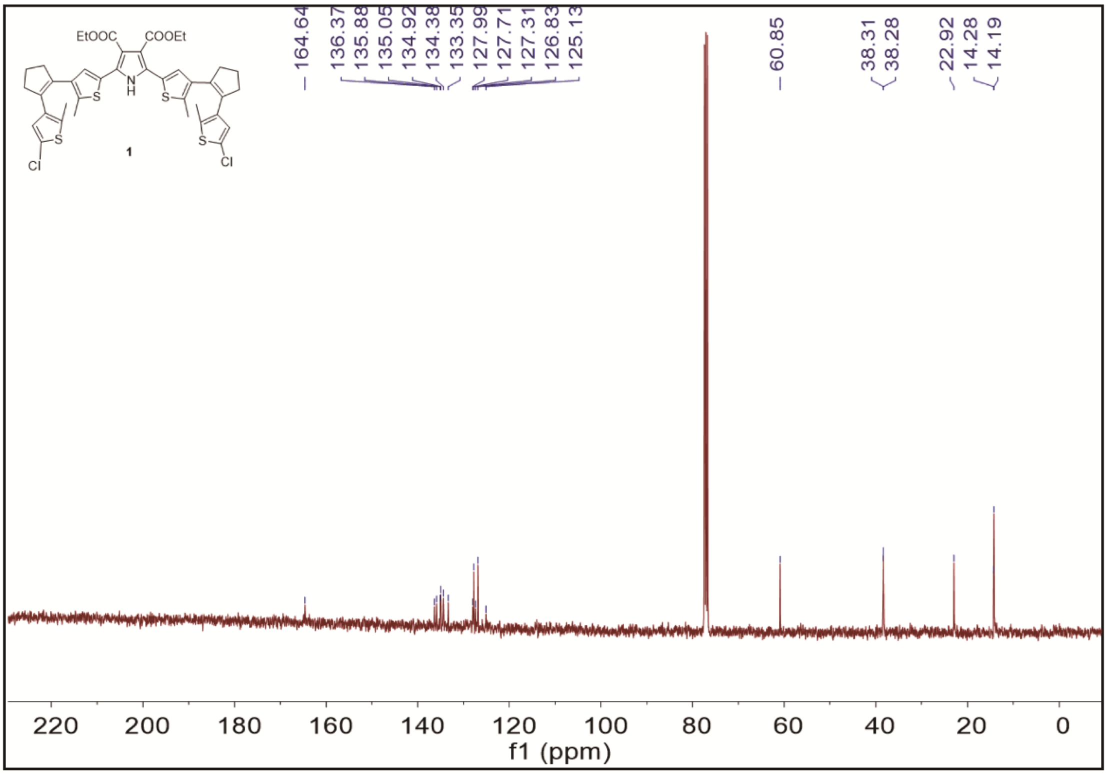

Supplementary Figure 26. ^{13}C NMR (100 MHz, $CDCl_3$) spectrum of compound **1**.

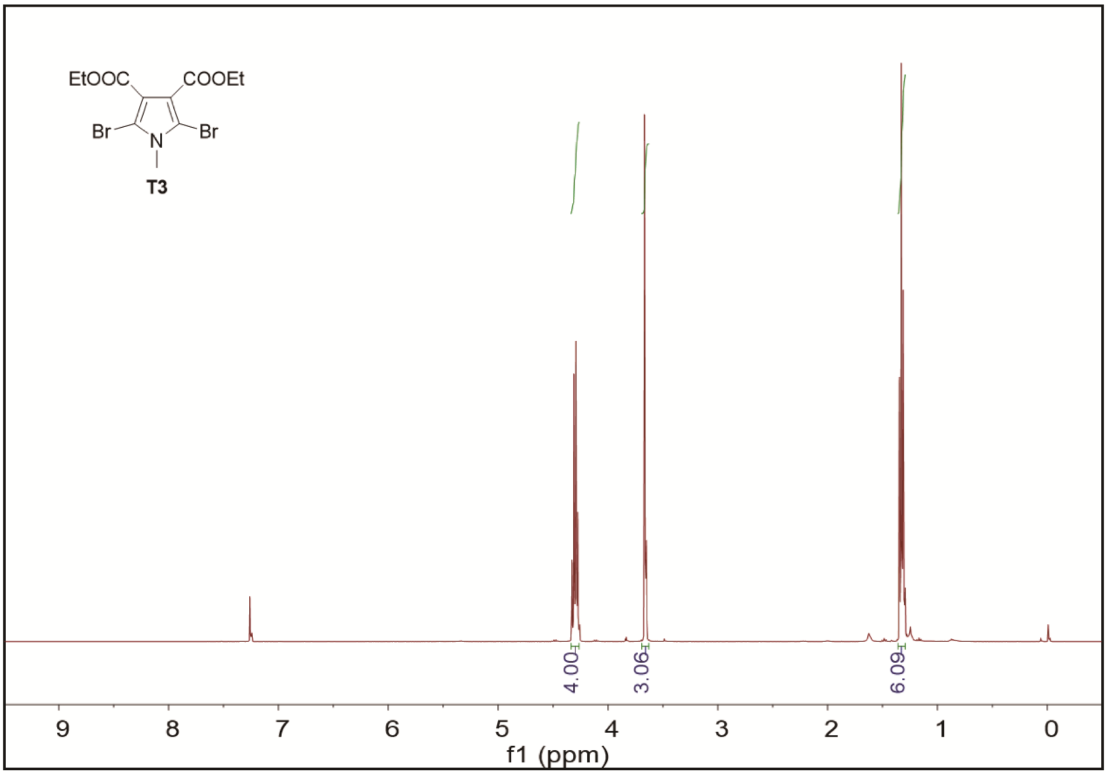

Supplementary Figure 27. ^{1}H NMR (400 MHz, $CDCl_3$) spectrum of compound **T3**.

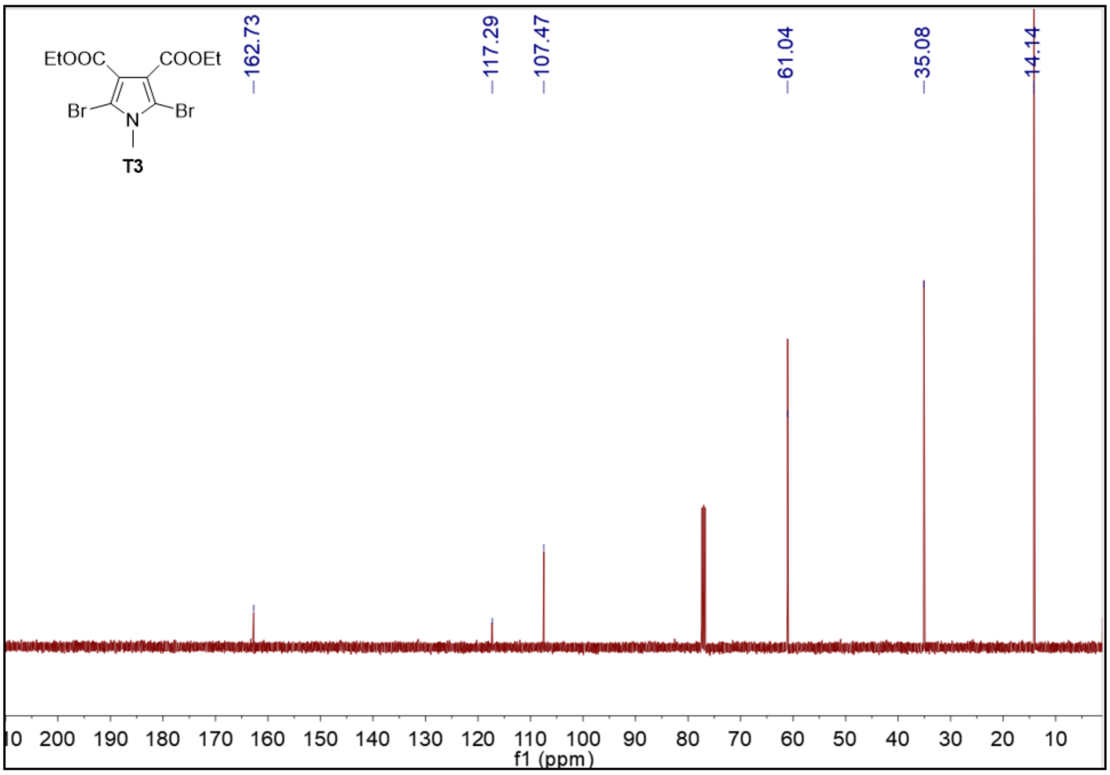

Supplementary Figure 28. ^{13}C NMR (100 MHz, $CDCl_3$) spectrum of compound **T3**.

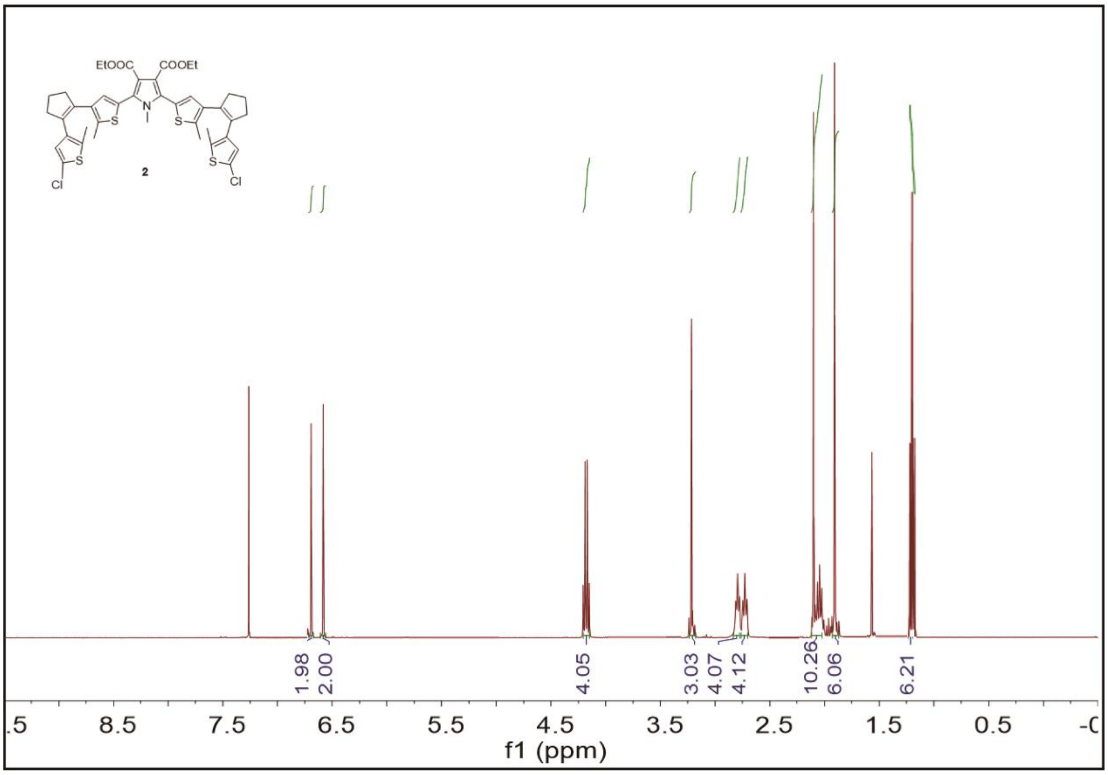

Supplementary Figure 29. ^{1}H NMR (400 MHz, $CDCl_3$) spectrum of compound **2**.

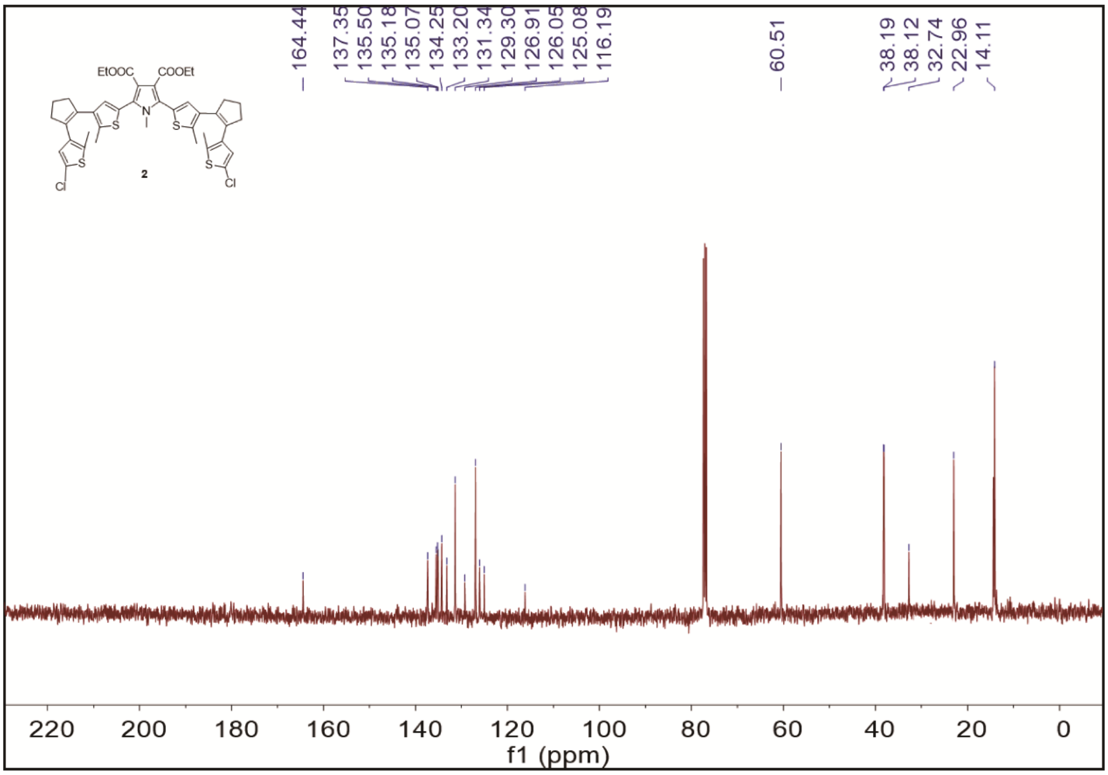

Supplementary Figure 30. ^{13}C NMR (100 MHz, $CDCl_3$) spectrum of compound **2**.

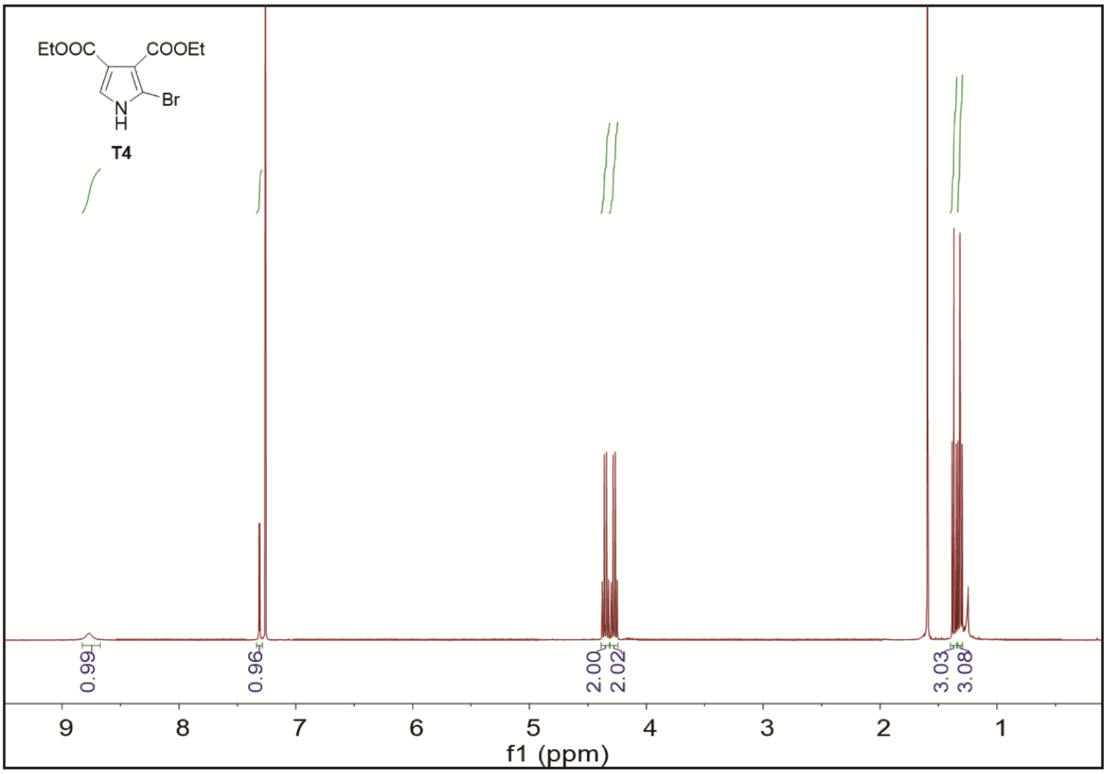

Supplementary Figure 31. ^{1}H NMR (400 MHz, $CDCl_3$) spectrum of compound **T4**.

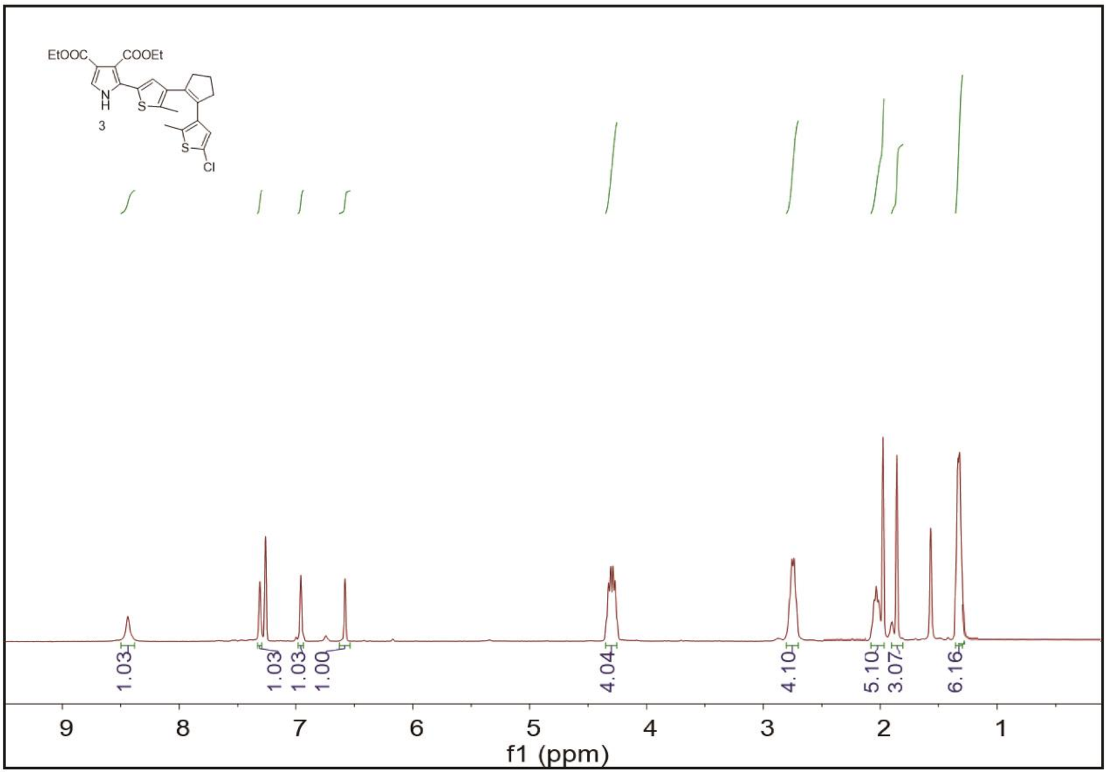

Supplementary Figure 32. ^{1}H NMR (400 MHz, $CDCl_3$) spectrum of compound **3**.

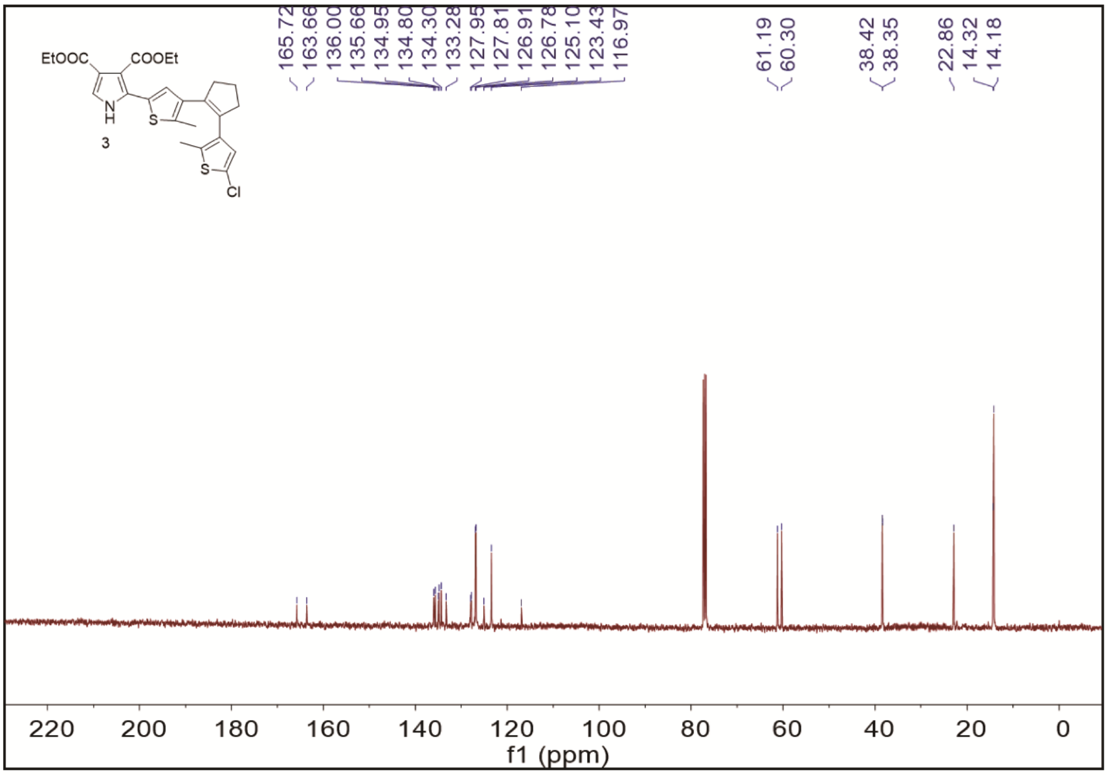

Supplementary Figure 33. ^{13}C NMR (100 MHz, $CDCl_3$) spectrum of compound **3**.

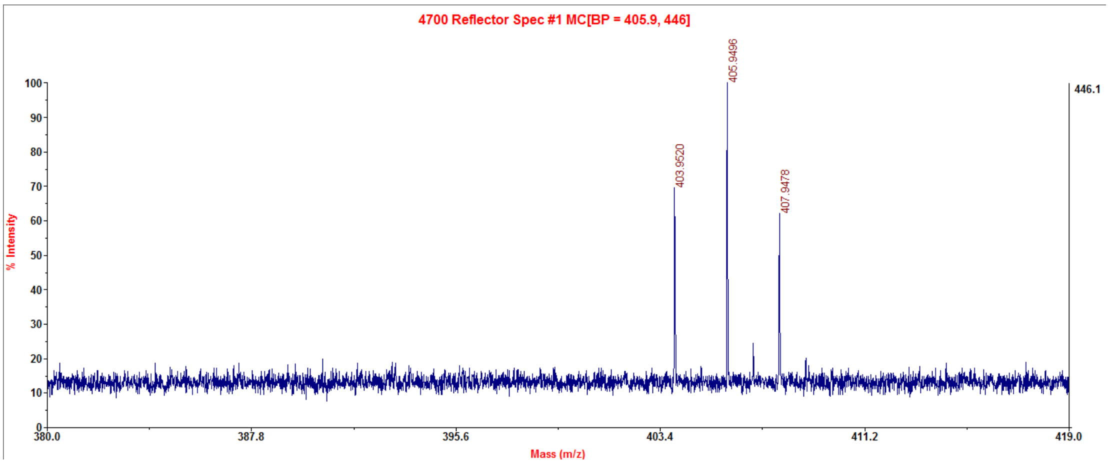

Supplementary Figure 34. MALDI-TOF-MS of compound **T3**.

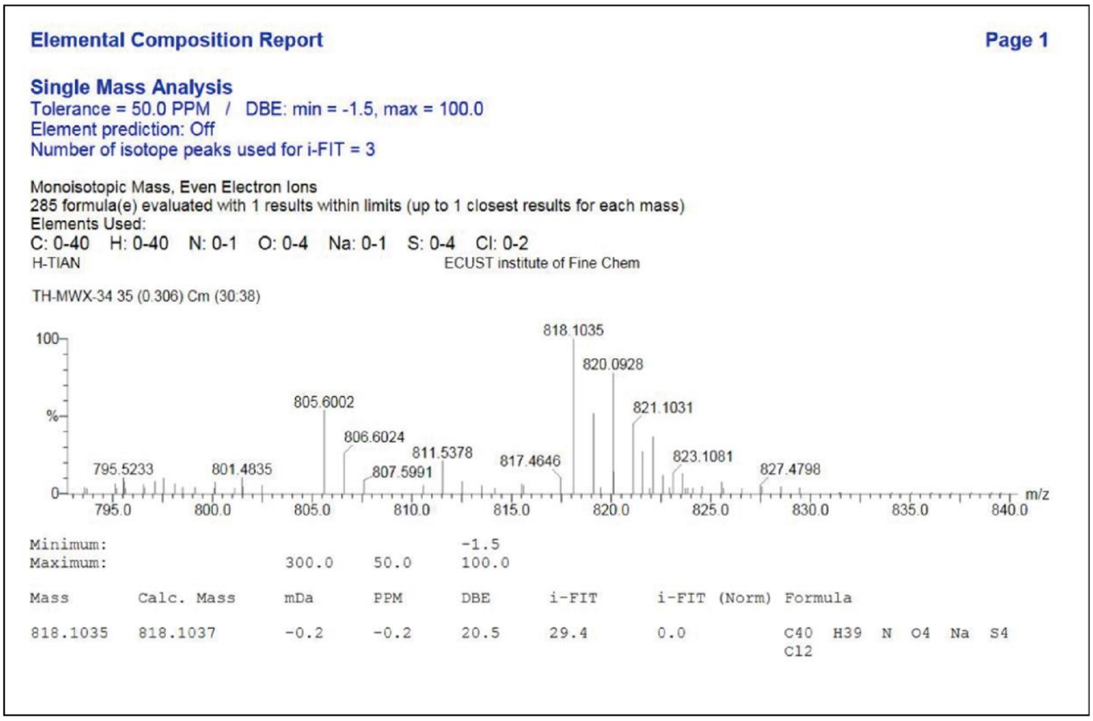

Elemental Composition Report — Page 1

Single Mass Analysis
Tolerance = 50.0 PPM / DBE: min = -1.5, max = 100.0
Element prediction: Off
Number of isotope peaks used for i-FIT = 3

Monoisotopic Mass, Even Electron Ions
285 formula(e) evaluated with 1 results within limits (up to 1 closest results for each mass)
Elements Used:
C: 0-40 H: 0-40 N: 0-1 O: 0-4 Na: 0-1 S: 0-4 Cl: 0-2
H-TIAN ECUST institute of Fine Chem

TH-MWX-34 35 (0.306) Cm (30:38)

	Calc. Mass	mDa	PPM	DBE	i-FIT	i-FIT (Norm)	Formula
Minimum:				-1.5			
Maximum:		300.0	50.0	100.0			
Mass	Calc. Mass	mDa	PPM	DBE	i-FIT	i-FIT (Norm)	Formula
818.1035	818.1037	-0.2	-0.2	20.5	29.4	0.0	C40 H39 N O4 Na S4 Cl2

Supplementary Figure 35. ESI-MS of compound **1**.

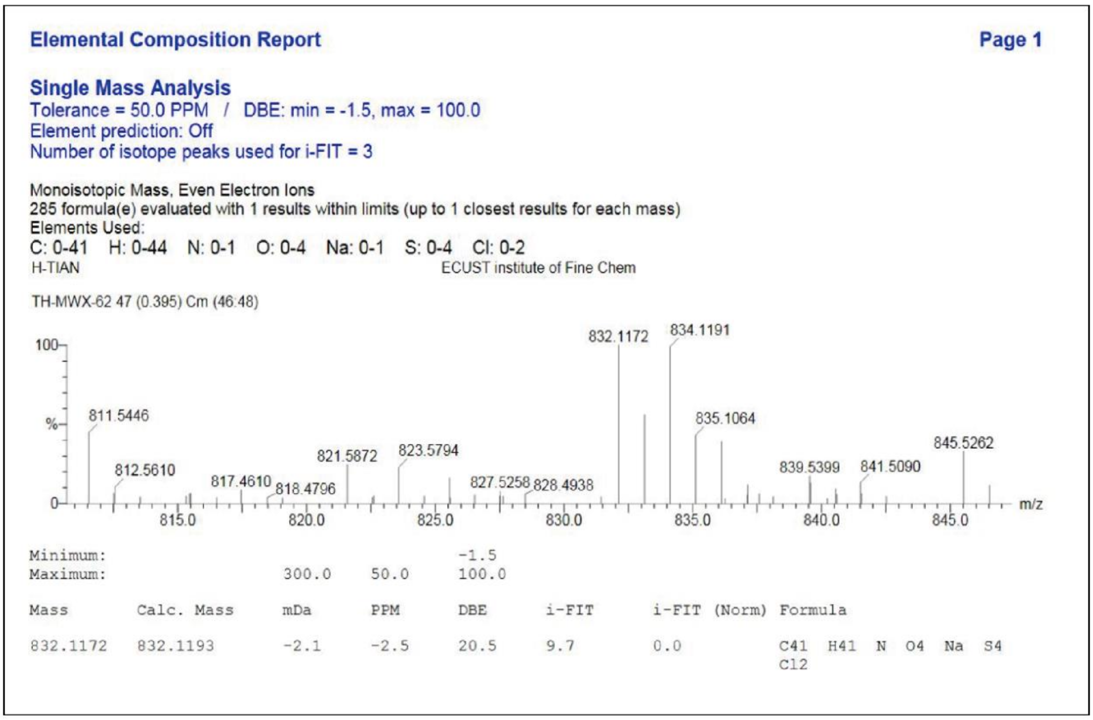

Supplementary Figure 36. ESI-MS of compound **2**.

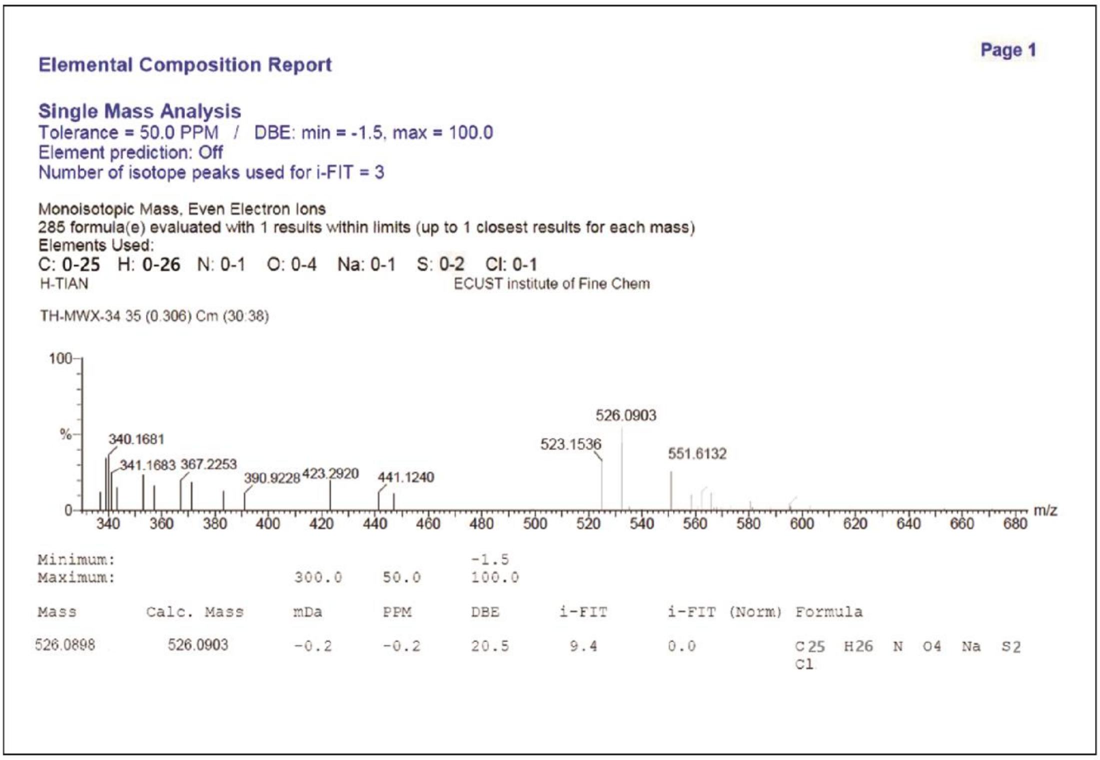

Supplementary Figure 37. ESI-MS of compound **3**.

Supplementary Tables

Supplementary Table 1. The components of the g-tensor for the ground doublet state of radical **1’** calculated by the UB3LYP/6-311++G(d,p) method within GIAO approximation.

Tensor component*	g^r	g^d	$g^{orb+SOC}$	g	$\bar{g}$
**radical 1’**					
xx	-1.18×10^{-4}	7.38×10^{-5}	1.85×10^{-3}	2.00406	2.00496
yy		1.53×10^{-4}	4.04×10^{-3}	2.00603	
zz		-1.09×10^{-4}	2.77×10^{-3}	2.00480	

* g^r– relativistic mass correction, g^d– diamagnetic correction, $g^{orb+SOC}$– orbital Zeeman and spin-orbit coupling contribution, g– total value of g-tensor diagonal component calculated as $g = g_e + g^r + g^d + g^{orb+SOC}$ (here g_e is a g value of the free electron 2.0023193), $\bar{g}$– average value of g-factor calculated as $\bar{g} = \frac{1}{3}(g_{xx} + g_{yy} + g_{zz})$.

Supplementary Table 2. The ^{1}H NMR data of compound **1** and its photostationary state based on measurements and calculations, respectively.

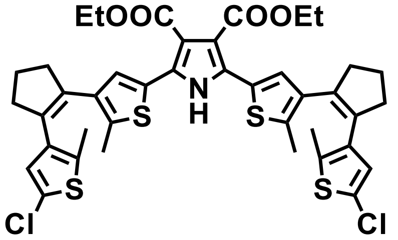

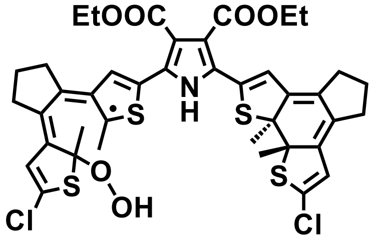

Identified protons	Measured	Calculated	Measured	Calculated
	Chemical shift (δ) in $CDCl_3$ (400 MHz ^{1}H NMR)			
ethyl-CH_3	1.32 (t)	1.14–2.03	1.31 (t)	1.18–1.57
thiophene-CH_3	1.87 (s)	2.02–2.16	1.87 (s)	1.76–2.45
cyclopentene-CH_2-	2.02–2.09 (m)	2.17–2.22	2.01–2.08 (m)	1.98–2.12
thiophene-CH_3	2.03 (s)	2.20–2.30	2.03 (s)	1.75–2.38
cyclopentene-CH_2-	2.74 (t)	2.98–3.03	2.74 (t)	2.86–3.13
cyclopentene-CH_2-	2.80 (t)	2.77–3.57	2.79 (t)	2.56–2.80
ethyl-CH_2-	4.28 (q)	3.80–5.01	4.28 (q)	4.06–4.62
thiophene-H	6.61 (s)	6.87	6.61 (s)	6.25
thiophene-H	7.00 (s)	7.53	7.00 (s)	7.92
pyrrole-H	8.09 (s)	8.64	8.09 (s)	7.93
peroxide-H			5.12 (s)	5.29

Supplementary Methods

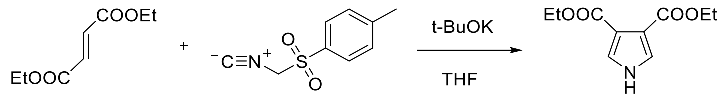

Synthesis of diethyl-*1H*-pyrrole-3,4-dicarboxylate. The synthesis of diethyl-*1H*-pyrrole-3,4-dicarboxylate was based on the literature procedure[1]. Under nitrogen atmosphere, the solution of diethyl fumarate (1.722 g, 10.0 mmol) and *p*-toluenesulfonylmethylisocyanide (1.952 g, 10.0 mmol) in 20.0 mL THF was added dropwise to the mixture of *t*-BuOK (2.244 g, 20.0 mmol) and 30.0 mL THF at 0 °C. The resulting white suspension turned to a yellow brown. The mixture was warmed to room temperature and stirred for 3 h, then poured into icy 1 M HCl aqueous solution (35.0 mL) to quench the reaction, extracted with ethyl acetate (30.0 mL×3), combined organic layers were washed with saturated $NaHCO_3$ solution (50.0 mL) and dried over anhydrous Na_2SO_4, filtrated, and concentrated. The residue was recrystallized with 6.0 mL ethyl acetate and 3.0 mL hexanes to give diethyl-*1H*-pyrrole-3,4-dicarboxylate (1.55 g, 73%) as an off-white solid.

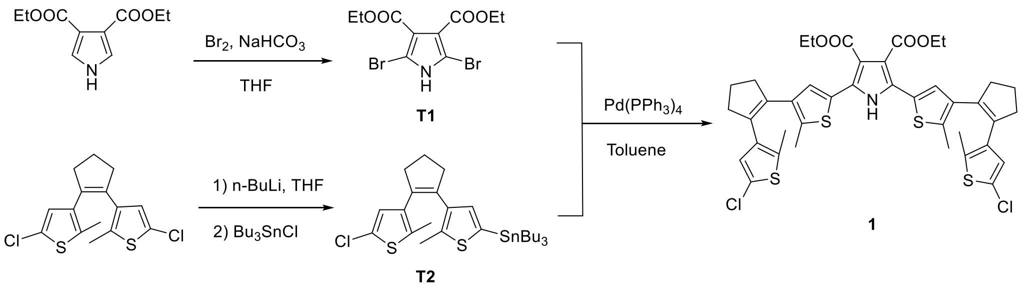

Synthesis of compound T1. The synthesis of compound **T1** was based on the literature procedure[2]. Under nitrogen atmosphere, Br_2 (2.47 g, 15.4 mmol) was added dropwise to the mixture of diethyl-*1H*-pyrrole-3,4-dicarboxylate (1.55 g, 7.35 mmol), $NaHCO_3$ (1.85 g, 22.1 mmol) and CH_2Cl_2 (115 mL) at -2 °C. The mixture was warmed to room temperature and stirred overnight, then poured into water (50.0 mL), extracted with CH_2Cl_2 (30.0 mL×3), combined organic layers were washed with saturated NaCl solution (50.0 mL) and dried over anhydrous Na_2SO_4, filtrated, and concentrated. The residue was purified by column chromatography (silica gel, CH_2Cl_2) to give the compound **T1** (2.10 g, 70%) as a pale-yellow solid. ^{1}H NMR (400 MHz, $CDCl_3$): δ 1.34 (t, J = 8.0 Hz, 6H), 4.32 (q, J = 8.0 Hz, 4H), 9.14 (s, 1H).

Synthesis of compound T2. Under nitrogen atmosphere, 1,2-bis(5-chloro-2-methyl-3-thienyl) cyclopentene (1.0 g, 3.04 mmol) was dissolved in anhydrous THF (20.0 mL) and *n*-BuLi (2.1 mL

of 1.6 M solution in hexane, 3.34 mmol) was added dropwise under nitrogen at -78 °C using a syringe. The mixture was stirred for 2 h at -78 °C and then the reaction mixture was quenched with tributyl tin chloride (1.08 mL, 3.95 mmol). The mixture was stirred for an additional hour at room temperature, concentrated and directly used in the next step without any purification.

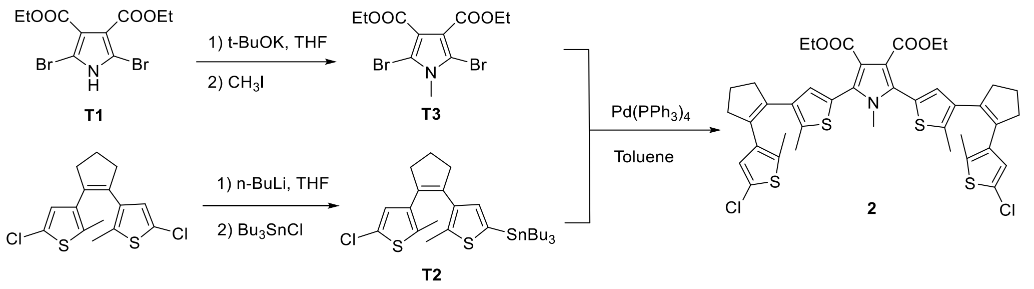

Synthesis of compound T3. Under nitrogen atmosphere, *t*-BuOK (0.370 g, 3.30 mmol) was added to the solution of compound **T1** (1.0 g, 2.70 mmol) in anhydrous THF (8.0 mL) at room temperature and stirred for 1 h. Then CH_3I (0.320 g, 3.30 mmol) was added dropwise and heated to 50 °C, and stirred for additional 2 h. After cooling to room temperature, the mixture was poured into water (20.0 mL), extracted with CH_2Cl_2 (30.0 mL×3), combined organic layers were washed with saturated NaCl solution (50.0 mL) and dried over anhydrous Na_2SO_4, filtrated, and concentrated. The residue was purified by column chromatography (silica gel, CH_2Cl_2/petroleum ether 2:1, v/v) to give the compound **T3** (0.70 g, 68%) as a white solid. ^{1}H NMR (400 MHz, $CDCl_3$): δ 1.33 (t, *J* = 8.0 Hz, 6H), 3.67 (s, 3H), 4.31 (q, *J* = 6.6 Hz, 4H). ^{13}C NMR (100 MHz, $CDCl_3$): δ 14.14, 35.08, 61.04, 107.47, 117.29, 162.73. HRMS (MALDI-TOF): $[M+Na]^+$ calcd. for $C_{11}H_{13}Br_2NO_4Na$, m/z: 403.9109; found, 403.9520.

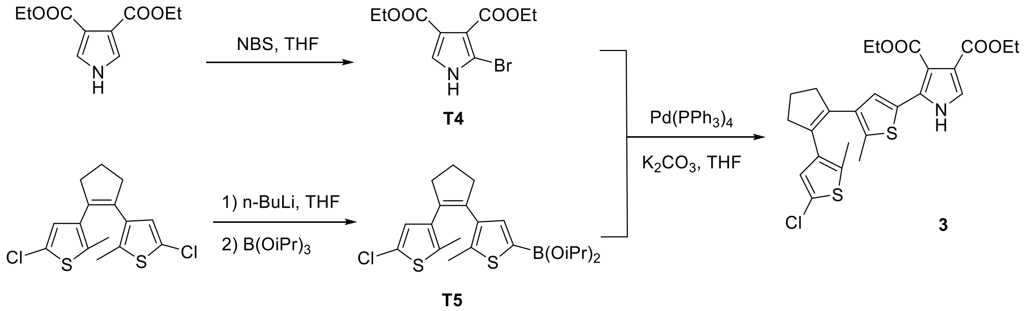

Synthesis of compound T4. The synthesis of compound **T4** was based on the literature procedure[3]. Under nitrogen atmosphere, the solution of NBS (0.460 g, 2.58 mmol) in anhydrous THF (15.0 mL) was added dropwise to the solution of diethyl-1H-pyrrole-3,4-dicarboxylate (0.545 g, 2.58 mmol)

in CH_2Cl_2 (20.0 mL) at -78 °C. Then the solution was warmed to 0 °C and stirred for additional 2 h before it was poured into water (20.0 mL). It was extracted with ethyl acetate (20.0 mL×3), combined organic layers were washed with saturated NaCl solution (30.0 mL) and dried over anhydrous Na_2SO_4, filtrated, and concentrated. The residue was purified by column chromatography (silica gel, ethyl acetate/petroleum ether 1:4, v/v) to give the compound **T4** (0.375 g, 50.1%) as a white solid. ^{1}H NMR (400 MHz, $CDCl_3$): δ 1.34 (t, J = 6.0 Hz, 3H), 1.37 (t, J = 6.0 Hz, 3H), 4.28 (q, J = 6.6 Hz, 2H), 4.35 (q, J = 6.6 Hz, 2H), 7.31 (d, J = 2.8 Hz, 1H), 8.77 (s, 1H).

Synthesis of compound T5. Under nitrogen atmosphere, 1,2-bis(5-chloro-2-methyl-3-thienyl) cyclopentene (0.247 g, 0.75 mmol) was dissolved in anhydrous THF (10.0 mL) and *n*-BuLi (0.52 mL of 1.6 M solution in hexane, 0.825 mmol) was added dropwise under nitrogen at -78 °C using a syringe. The mixture was stirred for 2 h at -78 °C and then the reaction mixture was quenched with triisopropyl borate (0.23 mL, 0.975 mmol). The mixture was stirred for additional 3 h at room temperature, and directly used in the next step without any purification.

Supplementary Note

Supplementary Note 1. Photochromic reaction quantum yields.

Photoreaction quantum yields of compound **1** irradiated at 313 nm were measured with the potassium ferrioxalate ($K_3[Fe(C_2O_4)_3]$) as the actinometer[4]. The solutions were prepared as follows: In the dark, 2.0 mL of a 0.006 M solution of ferrioxalate in 0.05 M H_2SO_4 in the cuvette (1 cm quartz cell) was irradiated with the investigated wavelength for 5 min. Subsequently, 0.5 mL of phenanthroline (0.1wt% in 0.5 M H_2SO_4/1.6 M NaOAc) was added to the cuvette and the absorbance at 530 nm was measured immediately. The irradiation time must be short in order to avoid more than 10% ferrioxalate decomposition. The moles of Fe^{2+} could be calculated by the formula Eq. (S1):

$$\text{moles of } Fe^{2+} = \frac{V_1 \times \Delta A(530\ \text{nm})}{V_2 \times l \times \varepsilon(530\ \text{nm})} \qquad \text{Eq. (S1)}$$

where V_2 = 2.0 mL, which is the irradiated volume; V_1 = 2.5 mL, which is the final volume after complexation with phenanthroline; l = 1 cm, which is the optical pathlength of the irradiation cell; ε(530 nm) is 11100 M^{-1} cm^{-1}, which is the molar extinction coefficient of the complex $Fe(phen)_3^{2+}$; ΔA is the optical difference in absorbance between the irradiated solution and that taken in the dark. Thus, the moles of photons (Nhv) absorbed by the irradiated solution per time unit are as follows:

$$\frac{Nh\nu}{t} = \frac{\text{moles of } Fe^{2+}}{\varphi_\lambda \times t \times F} \qquad \text{Eq. (S2)}$$

where φ_λ is the quantum yield of ferrous ion production at the irradiation wavelength, t is the time of irradiation, and F is the mean fraction of light absorbed by compound **1** solution, φ_λ is the quantum yield at the used irradiation wavelength (1.24 for 313 nm, 0.15 for 530 nm).

Then 2.0 mL of the compound **1** solution in degassed chloroform and acetonitrile in a cuvette was irradiated by the same investigated wavelength in the dark, respectively, and the absorbance at 530 nm was tested immediately. The concentration of compound **1** was 2.0×10^{-5} M. The photocyclization quantum yields can be calculated by the following formula Eq. (S3):

$$\varphi_x = \frac{\Delta A/\Delta t}{\left(Nh\nu/t\right) \times \varepsilon_x \times F_x} \qquad \text{Eq. (S3)}$$

where ΔA/Δt is the change of absorbance upon irradiation at detective wavelength, φ_x is the molar extinction coefficient at detective wavelength ($\varphi_{313\ nm}$ = 14000 M^{-1} cm^{-1}, $\varphi_{530\ nm}$ = 11200 M^{-1} cm^{-1} for compound **1** in chloroform; $\varphi_{313\ nm}$ = 13500 M^{-1} cm^{-1}, $\varphi_{530\ nm}$ = 11000 M^{-1} cm^{-1}) and Fx is the

mean fraction of light absorbed, the value of which is $1–10^{-A}$.

Based on the above methods, the photocyclization quantum yields and cycloreversion quantum yields of compound **1** were determined to be 48% at 313 nm in chloroform, and 31% at 313 nm in acetonitrile, respectively.

Supplementary References

1. Francisco, F., Juliana, M., Beatriz, H, K., Timothy, J. & Kleber, T. Synthesis and photophysical studies of a chlorin sterically designed to prevent self-aggregation. *Dyes Pigments* **92**, 153–159 (2013).
2. Algi, M. P., Öztaş, Z. & Algi, F. Triple channel responsive Cu^{2+} probe. *Chem. Comm.* **48**, 10219–10221 (2012).
3. Ho, I.-T. et al. A hybrid macrocycle with a pyridine subunit displays aromatic character upon uranyl cation complexation. *J. Am. Chem. Soc.* **136**, 4281–4286 (2014).
4. Murov, S, L. Handbook of photochemistry. 1973.
